# Comparative analysis of bioenergetic pathways in Medusozoa based on integrated genomic, transcriptomic and proteomic data

**DOI:** 10.3389/fphys.2026.1932200

**Published:** 2026-10-05

**Authors:** Edgar Gamero-Mora, Legna Gabriela Pasos-Ramirez, Julio Cesar Barcelo-Navarro, Adriana Muhlia-Almazan

**Affiliations:** 1 Department of Biological Sciences, University of Texas at El Paso, El Paso, TX, United States; 2 Laboratory of Bioenergetics and Molecular Genomics, Centro de Investigacion en Alimentacion y Desarrollo, A.C. (CIAD), Hermosillo, Sonora, Mexico

**Keywords:** anaerobic metabolism, energetic metabolism, jellyfish, mitochondria, phosphagen kinases, *Stomolophus* sp.2

## Abstract

**Introduction:**

Jellyfish possess the biochemical and physiological capabilities that enable them to survive from the beginning of the animal era to the impacts of global warming (ocean warming and deoxygenation) on the seas today. Despite their confirmed ability to bloom, significant gaps remain about the molecular basis of specific physiological responses, and limited data allow us to understand the metabolic pathways that enable medusozoans to transform energy and successfully cope with the challenges the marine environment imposes.

**Methods:**

This research integrates and describes prior knowledge (genomic and transcriptomic information) and experimental data (a mitochondrial proteome) to compare, analyze, and reconstruct the major bioenergetic pathways of Medusozoa.

**Results:**

From the conserved glycolytic enzymes to the novel and distinctive molecules enabling jellyfish to build large energy reserves, this manuscript describes the presence of alternative enzymes composing the mitochondrial branched respiratory chain, anaerobic metabolism, and the phosphagen systems in the four classes of true jellyfish.

**Discussion:**

These results may contribute, from an energetic perspective, to explain the physiological ability of jellyfish to cope with environmental changes by increasing their populations and occupying new environments.

## 1 Introduction

It is well known that the increase in ocean oxygen levels during the Neoproterozoic era is closely linked to the greater efficiency of animals in using oxygen to produce energy and to the consequent large diversity of marine species (Lane, 2022). Just as most of the animal kingdom depends on oxygen for survival, some ectotherms, including species of nematodes, crustaceans, mollusks, echinoderms, and some cnidarians, display enhanced tolerance to hypoxia and oxidative stress, being well adapted to produce the energy they need to survive (Moreira et al., 2016; Wang et al., 2017).

Energetic metabolism is central to life, as it allows the generation of chemical energy from food, which is essential for a species’ survival. Even though the metabolic pathways and molecules involved in energy generation have been intensively studied in model species (Tian et al., 2017), evidence shows that each species’ energetic metabolism and mitochondrial functions are adapted to its living conditions and environmental challenges (Heine and Wood, 2020).

Cnidarians in the Medusozoa subphylum include species that inhabit coastal ecosystems frequently affected by global warming, which primarily alters seawater oxygen concentrations and raises sea temperatures (Condon et al., 2001; Aljbour et al., 2017; Arossa et al., 2021). Under challenging conditions, some species have shown metabolic responses to cope with environmental stress, including adjusted metabolic rates and energy demands, suggesting metabolic plasticity, as well as sustained mitochondrial function to produce energy (Sokolova et al., 2012; Nevarez-Lopez et al., 2024).

The ability to withstand high temperatures and tolerate hypoxic conditions in some of the most studied medusozoans is likely related to their evolutionary history. However, the efficiency of oxygen use and their mitochondrial-specific characteristics have suggested specific adaptations for energy transformation (Nevarez-Lopez et al., 2020; 2024). Understanding the bioenergetics of these species at the molecular level will allow us to explain the basis of metabolic responses that give them an advantage over many other marine invertebrates under low-oxygen conditions or in fluctuating environments, contributing to their ability to form blooms and expand into new marine environments in recent decades (Schiariti et al., 2014; Bosch-Belmar et al., 2020; Fernandez-Alias et al., 2021; Rowe et al., 2022; Lopez-Martinez et al., 2023).

Previous evidence confirms that adult jellyfish at the medusa stage have higher energy requirements than their small benthic polyps, which have lower energy requirements due to their low metabolic rates (Urias-Padilla, 2021). However, the basic molecular structure underlying these species’ bioenergetic pathways, including the highly conserved molecules and the enzymes conferring specific biochemical and physiological abilities in jellyfish, has not been comprehensively analyzed or described to date.

These results will allow us to identify the molecules and enzymes involved in both aerobic and anaerobic pathways and to assess their similarities and differences relative to other well-studied animal models, thereby improving our understanding of the molecular mechanisms underlying physiological performance and environmental resilience in Medusozoa.

## 2 Materials and methods

### 2.1 Database description and the energetic metabolic pathways reconstruction

The molecular basis of energetic metabolism in medusozoans (hereinafter referred to as jellyfish in this manuscript) was examined using publicly available genomic and transcriptomic datasets representing the four Medusozoa classes (Cubozoa, Hydrozoa, Scyphozoa, and Staurozoa). **Table 1** lists the assembled transcriptomes/genomes from 13 medusozoans that represent the major subclasses, orders, or suborders across the four classes within the subphylum Medusozoa, and the recently published transcriptome of *Stomolophus* sp.2.

**Table 1:** Taxonomic classification of medusozoan species included in the analysis of deduced proteins related to energy metabolism.

Class	Subclass	Order	Suborder	Family	Genus	Species
Cubozoa		Carybdeida		Tripedaliidae	Copula	*C. sivickisi* (PRJNA498767; T)
		Chirodropida		Chirodropidae	Chironex	*C. yamaguchii* (PRJNA498204; T)
Hydrozoa	Hydroidolina	Anthoathecata	Filifera	Hydractiniidae	Hydractinia	*H. symbiolongicarpus* (PRJNA237004; T)
			Aplanulata	Hydridae	Hydra	*H. vulgaris* (GCF_038396675; G)
		Leptothecata	Sertularioidea	Sertulariidae	Dynamena	*D. pumila* (PRJNA526875; T)
				Campanulariidae	Clytia	*C. hemisphaerica* (GCF_902728285; G)*
		Siphonophorae	Physonectae	Agalmatidae	Nanomia	*N. bijuga* (GCA_032273505; G)
Staurozoa		Stauromedusae	Amyostaurida	Craterolophidae	Craterolophus	*C. convolvulus* (PRJEB19215; T)
			Myostaurida	Haliclystidae	Haliclystus	*H. sanjuanensis* (PRJEB19215; T)
Scyphozoa	Coronamedusae	Coronatae		Atollidae	Atolla	*A. vanhoeffeni* (PRJNA263637; T)***
	Discomedusae	Rhizostomeae	Dactyliophorae	Rhizostomatidae	Rhopilema	*R. esculentum* (GCF_013076305; G)
	Discomedusae	Rhizostomeae	Dactyliophorae	Stomolophidae	Stomolophus	*Stomolophus* sp.2 (PRJNA851814; T)
			Kolpophorae	Cassiopeidae	Cassiopea	*C. xamachana* (Cxam.augustus.1.3; G)**
		Semaeostomeae		Pelagiidae	Chrysaora	*C. quinquecirrha* (PRJNA555711; T)

The NCBI BioProject accession number is indicated in the last column. Letter “T” denotes transcriptomic datasets, and “G” indicates genomic datasets. Asterisks indicate datasets not available in NCBI but retrieved from other repositories. Data source links:.

*https://www.ebi.ac.uk/ena/browser/view/GCA_902728285.1.

**https://github.com/josephryan/Ohdera_et_al_2018/tree/master/AA_Files.

***https://bitbucket.org/caseywdunn/cnidaria2014-assemblies/src/master/Cnidaria_Transcriptomes/.

Each metabolic pathway was first inferred and reconstructed for each jellyfish species based on the confirmed presence of genes or transcripts encoding proteins and enzymes with the specific conserved characteristics of molecules that integrate each canonical metabolic pathway. The analysis included key energy-transformation pathways, such as aerobic metabolism (glycolysis, the tricarboxylic acid cycle, the electron transport chain, and oxidative phosphorylation) and anaerobic pathways (lactate production and the phosphagen systems that play a central role in short-term ATP buffering outside mitochondria).

The transcriptome of *Stomolophus* sp.2 (Gamero-Mora et al., 2025; GenBank Bioproject PRJNA851814) and the mitochondrial proteome database (unpublished) obtained by our group were then integrated into the analysis to confirm data, fill gaps, and provide a detailed reference for the composition of each energy-related pathway in this manuscript.

The absence of a specific gene or transcript was carefully analyzed and reviewed; then it was only suggested to be absent whether it was not detected in any of the analyzed databases, since low quality of databases or low transcript abundance may also explain it.

The analytical workflow including the bioinformatic pipeline used to reconstruct the bioenergetic metabolic pathways of Medusozoa is summarized in the **Supplementary Material** (**Supplementary Material 1**).

All protein searches followed the same general bioinformatic workflow. Reference protein or nucleotide sequences were selected from public databases according to the analyzed metabolic pathway and used as queries in local BLAST searches against available genomic, transcriptomic, or predicted protein datasets. BLASTn was performed when nucleotide assemblies were available, and BLASTp was used for predicted protein datasets. The ten hits with the lowest E-values were retained for further evaluation. When nucleotide sequences were recovered, the corresponding coding regions were translated into protein sequences using the appropriate open reading frame inferred with ExPASy Translate tool.

(https://web.expasy.org/translate/). Candidate proteins were subsequently confirmed using the BLAST algorithm (http://www.ncbi.nlm.nih.gov/blast). The specific conserved protein domains and functional motifs were identified using ExPASy-PROSITE (https://prosite.expasy.org/), the NCBI Conserved Domain Database (https://www.ncbi.nlm.nih.gov/Structure/cdd/cdd.shtml), and HMMER (https://www.ebi.ac.uk/Tools/hmmer/search/phmmer).

The presence or absence of a signal peptide to confirm the predicted mitochondrial or cytosolic location of alternative enzymes was predicted by TargetP2.0 (https://services.healthtech.dtu.dk/services/TargetP-2.0/), MuLocDeep (https://www.mu-loc.org/), and DeepLoc2.1 (https://services.healthtech.dtu.dk/services/DeepLoc-2.1/).

Multiple sequence analyses were performed for specific enzymes to compare and confirm the general and conserved characteristics of deduced proteins among medusozoans or to compare them with enzymes from vertebrate species. Genomic and transcriptomic datasets used in the analyses are identified by their accession numbers or repository identifiers in **Table 1**. Reference sequences used for individual protein searches are identified by their accession numbers in the corresponding methodological sections. Protein sequences were aligned using the Clustal Omega algorithm with default parameters.

Due to the number and complexity of genes involved in each metabolic pathway, different strategies were used as follows:

#### 2.1.1 Glycolysis and the tricarboxylic acid cycle

The 10 and 8 enzymes that catalyze the reactions involved in glycolysis and the TCA cycle, respectively, were identified. Three to four representative enzymes from each pathway were examined across the 13 jellyfish datasets to evaluate their conservation among Medusozoa. The analyses focused on enzymes catalyzing regulatory or irreversible steps, as well as selected enzymes representing central pathway functions and core subunits of multimeric enzyme complexes. For these highly conserved metabolic enzymes, reviewed Swiss-Prot entries from model organisms were used as queries. The glycolysis analysis used accessions Q9NFT7, P52034, P13706 and O62619. The TCA cycle analysis used accessions Q9VWH4, P11498, and Q9W401.

#### 2.1.2 Electron transport chain and oxidative phosphorylation

Because oxidative phosphorylation comprises a large number of proteins distributed among five multimeric mitochondrial complexes, a representative assessment of pathway conservation was performed using five species representing the four recognized medusozoans classes: *Copula sivicksi* (Cubozoa), *Haliclystus sanjuanensis* (Staurozoa), *Stomolophus* sp.2 (Scyphozoa), *Hydra vulgaris* (Hydrozoa), and *Rhopilema esculentum* (Scyphozoa). Transcriptome assemblies of *C. sivickisi*, *H. sanjuanensis*, and *Stomolophus* sp.2 were translated and annotated using TransDecoder and Trinotate, and candidate proteins were identified from the annotation reports. For *H. vulgaris* and *R. esculentum*, available KEGG genomic annotations were used. All protein subunits included in the KEGG oxidative phosphorylation pathway (hsa00190) were evaluated. Protein identities were confirmed following the general workflow described above.

The presence/absence of four alternative mitochondrial enzymes in the jellyfish branched respiratory chain, previously identified in *Stomolophus* sp.2 (Nevarez-Lopez et al., 2024), was investigated across all transcriptomic and genomic datasets, following the general bioinformatic workflow described above.

#### 2.1.3 Identification and characterization of specific and novel proteins

The anaerobic glycolytic pathway was studied by analyzing L-lactate dehydrogenase (L-LDH), the enzyme that mediates the conversion of pyruvate to lactate in most metazoans. A well-characterized L-lactate dehydrogenase sequence (Swiss-Prot: Q95028) was used as the reference query to search all genomic and transcriptomic datasets following the workflow described above. The evolutionary relationships among the recovered LDH sequences were examined using phylogenetic inference, as described below.

In addition, two putative phosphagen systems were explored by analyzing the diversity of phosphagen kinases in jellyfish, the two well-characterized enzymes in metazoans, arginine kinase (AK) and creatine kinase (CK). Representative arginine kinase (EZ016454.1) and creatine kinase (XM_065195832.1) sequences from Cnidaria were used as reference queries to search for homologous phosphagen kinases across all datasets. After multiple homologous sequences were recovered for phosphagen kinases, phylogenetic analyses were performed to assess their relationships and facilitate sequence classification.

The **Supplementary Table 1** lists the reference sequences and the GenBank accession numbers used in the phylogenetic analyses of LDH and phosphagen kinases. Appropriate outgroup taxa were selected based on the protein family analyzed.

### 2.2 The mitochondrial proteome of jellyfish *Stomolophus* sp.2.

Some specific mitochondrial-predicted proteins of jellyfish were confirmed by peptide identification in the mitochondrial proteome of *Stomolophus* sp.2. Mitochondria were isolated from the bell of three adult jellyfish of the species *Stomolophus* sp.2 following the differential centrifugation method previously described by Nevarez-Lopez et al. (2024). Protein extraction, quantification, and trypsin digestion were performed as described by Caña-Bozada et al. (2024). Peptides were analyzed by Nano LC-MS/MS, using a C18 precolumn (3 μm, 75 μm × 20 mm, Dionex) at 3 μL/min flow rate and resolved on a C18 column (2 μm, 75 μm × 250 mm) with a flow rate of 300 nL/min connected to an UHPLC system (UltiMate 3000, Dionex, Sunnyvale, CA) and interfaced with a Fusion™ Tribrid™Orbitrap (Thermo-Fisher Scientific, San Jose, CA) mass spectrometer (Thermo-Fisher Scientific, San Jose, CA) as reported by Bojorquez-Velazquez et al. (2022).

Protein identification was performed on the raw file and processed with Proteome Discoverer 3.0 (PD, Thermo Fisher Scientific) using the SEQUEST HT search engine against an in-house *Stomolophus* sp.2 protein database that resulted from the translated transcriptome of our group (Gamero-Mora et al., 2025). Search parameters included full-tryptic protease specificity, allowing two missed cleavages; carbamidomethylation of cysteine (+57.021 Da) was set as a fixed modification. Methionine oxidation (+15.995 Da) and deamidated asparagine, glutamine, and arginine (+0.984016) were selected as variable modifications. Mass tolerances of ± 20 ppm and ±0.1 Da were applied for precursor and fragment ions, respectively. The resulting peptide hits were filtered to a maximum FDR of 1% using the Percolator algorithm.

### 2.3 Phylogenetic relationships

Phylogenetic analyses were performed to confirm the protein’s homology and relations among medusozoans using transcripts encoding putative proteins involved in energy metabolism. The phylogenetic framework includes high-quality annotations to ensure their correct identification as enzymes participating in the confirmed energy metabolism pathway. Analyses were conducted using IQ-TREE v2.0.7 (Nguyen et al., 2015) and the ModelFinder implementation (Kalyaanamoorthy et al., 2017) for model selection. The ultrafast bootstrap (1,000 pseudoreplicates; Hoang et al., 2018) and SH-aLRT (1,000 iterations; Guindon et al., 2010) were used to assess the statistical support for phylogenetic clades. The resulting trees were visualized and edited in iTOL v7 (https://itol.embl.de/). The alignments used as input for the phylogenetic analyses were obtained using MAFFT (Katoh and Standley, 2013).

## 3 Results

Representative genomic and transcriptomic datasets from 14 medusozoan species spanning the four recognized classes enabled the reconstruction of the principal aerobic and anaerobic energy metabolism pathways. The profiles of genes, transcripts, and their deduced and identified proteins that compose energetic pathways were similar but not identical among different jellyfish classes. Various lineage-specific differences and previously unrecognized proteins are described in the following sections. All these data are primarily based on comparative bioinformatic analyses and do not include direct experimental validation.

### 3.1 The jellyfish aerobic metabolic pathways

#### 3.1.1 Glycolysis

Except for *A. vanhoeffeni*, the analysis confirmed that the three regulatory enzymes, hexokinase (HK), phosphofructokinase (PFK), and pyruvate kinase (PK), are encoded by at least one transcript in all analyzed medusozoans (**Figure 1**). The HK deduced protein in medusozoans include the two conserved signatures of HKs, a small subdomain near the N-terminus (the sugar-binding site) and a large subdomain near the C-terminus of the protein. The conserved amino acids in the binding sites of HKs and the high number of glycine residues conferring flexibility to the enzyme were detected in jellyfish sequences (Kuser et al., 2000), as indicated in the multiple alignment in (**Supplementary Material 2**).

**Figure 1:**
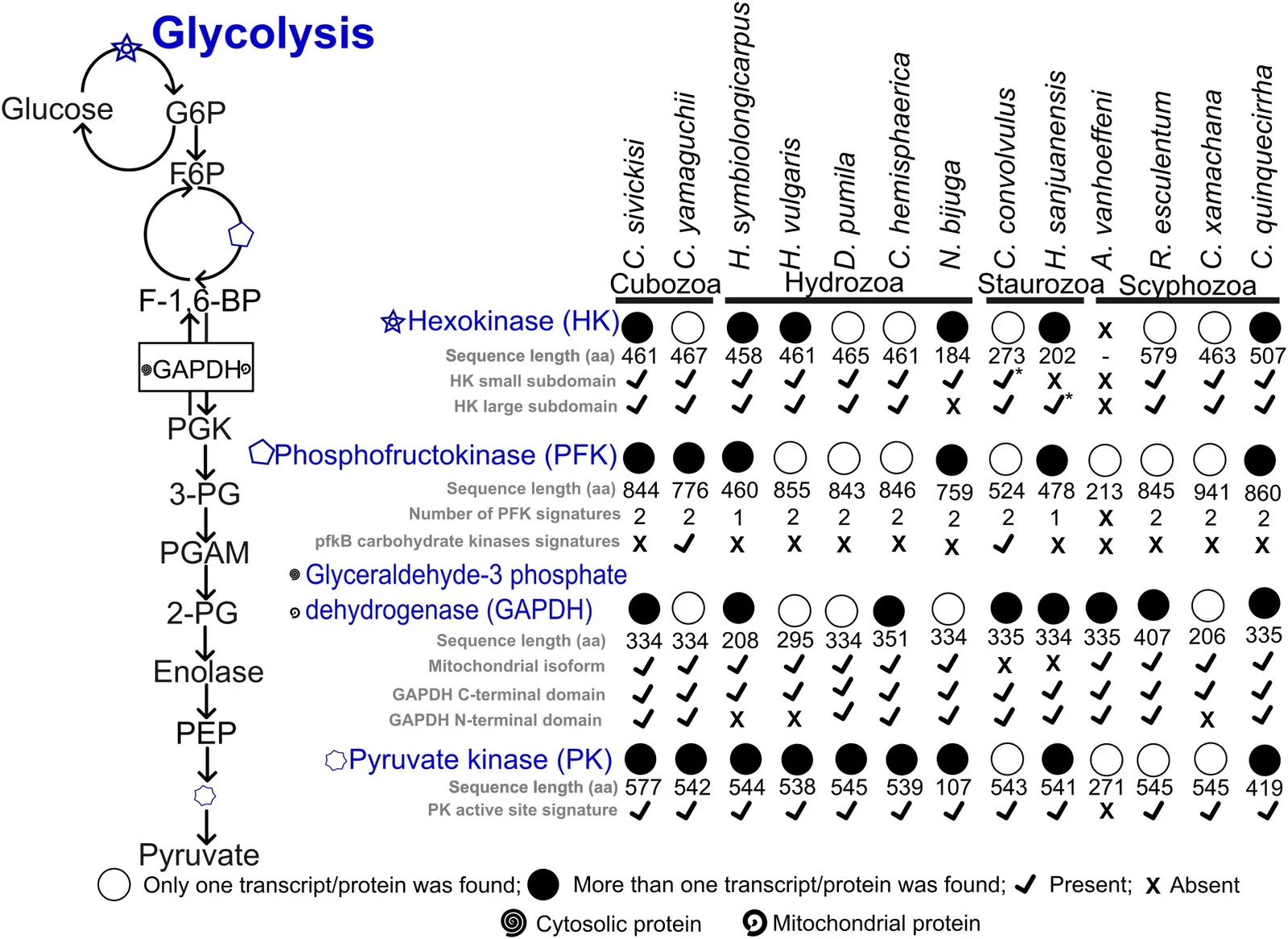
The four key glycolytic enzymes of medusozoans from different taxonomic classes. The left panel illustrates the enzyme’s position in the metabolic pathway, with each analyzed enzyme marked with a symbol. The right panel indicates across medusozoan species each of the analyzed enzymes including one single transcript (white circle) or multiple transcripts (black circle); the protein sequence length (number of residues); and the presence/absence of conserved signatures and subdomains based on NCBI’s Conserved Domain Search. *Indicates the presence of subdomains with partial sequences but with confirmed identity. The specific mitochondrial or cytosolic predicted localization of isoforms is also indicated. Absent molecules were labeled when the analysis of the top-10 hits in the BLAST search does not allow to confirm the identity of any of the molecules analyzed.

The presence of PFK was confirmed by a single transcript encoding a muscle-type-like PFK in most analyzed species. The PFK protein of Meduzosoans is longer (800–850 residues) compared to the human PFKs (780 residues), it includes it includes the eukaryotic gene-duplicated characteristics and the structural organization of mammalian PFKs, such as the conserved motif [RK]-x(4)-[GAS]-H-x-[QL]-[QR]-[GS] -[GF]-x(5)-[DE]-[RL] (Shullia et al., 2019; **Supplementary Material 3**). The jellyfish bell also expresses a transcript encoding a PK, which shares the conserved sites exclusive to muscle-like isoforms (M1 and M2) of vertebrates, with an active site signature that includes variations from the canonical conserved site of PKs and identity percentages of 52- 61% to both isoforms, respectively (Enriqueta-Muñoz and Ponce, 2003; de Klerk et al., 2012; **Supplementary Material 4**).

A significant difference in jellyfish compared to mammalian glycolytic enzymes is the presence of a different phosphoglycerate mutase (PGAM) isoform that catalyzes the reversible conversion of 3-phosphoglycerate to 2-phosphoglycerate. Jellyfish datasets confirmed the presence of a 2,3-bisphosphoglycerate-independent phosphoglycerate mutase isoform (iPGM, EC 5.4.2.12) instead of the cofactor-dependent isoform (EC 5.4.2.11) found in mammals.

Two glyceraldehyde-3-phosphate dehydrogenases (GAPDH), cytosolic and mitochondrial isoforms, were confirmed in the transcriptome and proteome of *Stomolophus* sp.2; both proteins share 24% identity, even though it is expected that both enzymes catalyze the same reaction, the structural differences and their location suggest different functions requiring experimental validation (Brown et al., 1994; **Supplementary Material 5**).

All jellyfish species display a well-conserved glycolytic pathway, with differences in the number, types, and locations of enzyme isoforms that catalyze the same specific reactions.

#### 3.1.2 The tricarboxylic acid cycle

In addition to the eight enzyme sequences that participate in the TCA, one of its regulatory enzymes, pyruvate carboxylase (PC), was confirmed in jellyfish; its functional characteristics, including the enzyme-specific conserved motifs and catalytic sites, were analyzed. **Figure 2** shows the homology-based search for key enzymes, including pyruvate carboxylase (PC), citrate synthase (CS), and isocitrate dehydrogenase subunit 3 alpha (IDH3A).

**Figure 2:**
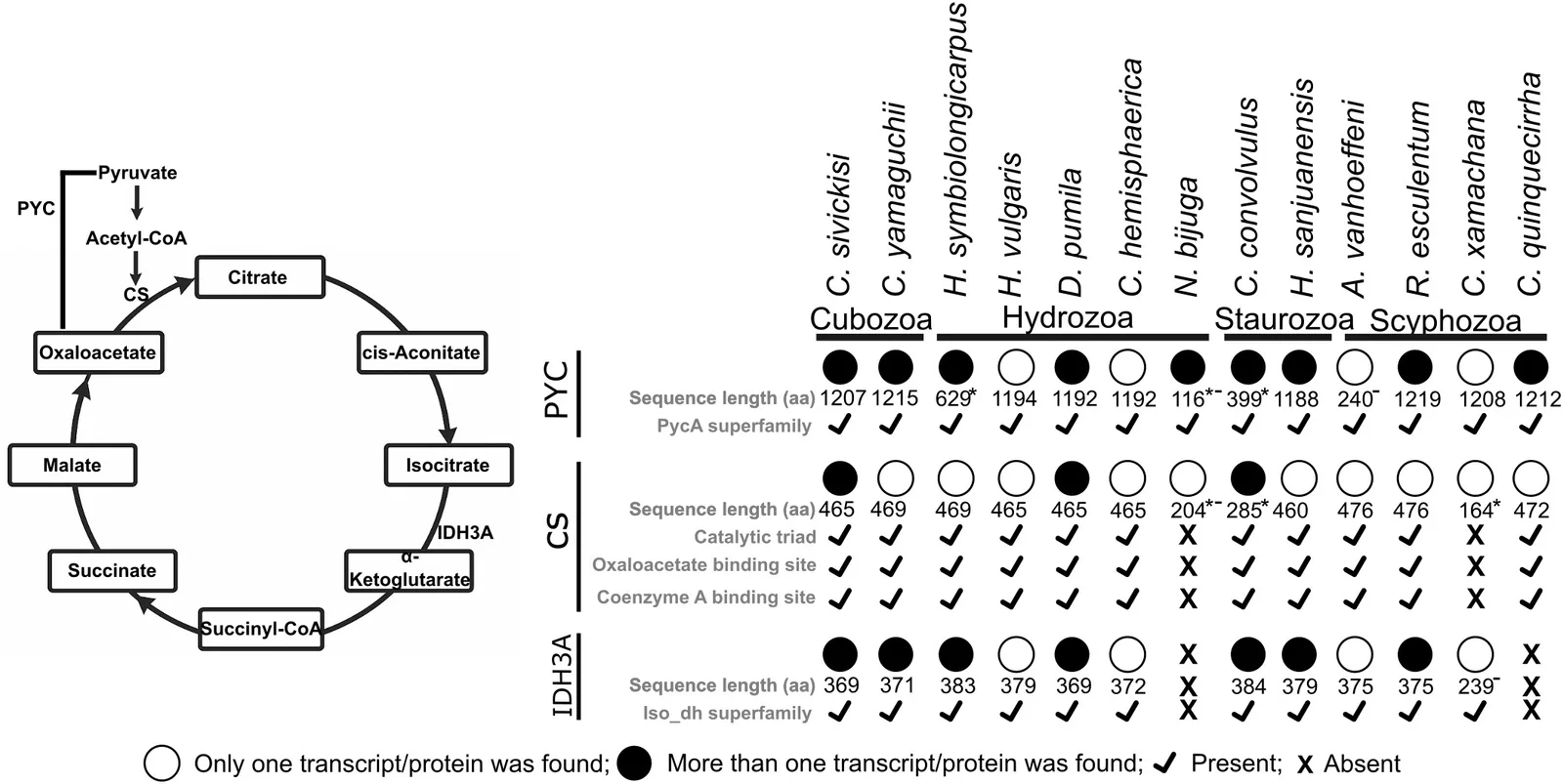
The three key enzymes in the TCA of medusozoans from different taxonomic classes. Pyruvate carboxylase (PC), citrate synthase (CS), and isocitrate dehydrogenase 3 alpha subunit (IDH3A). The left panel illustrates the enzyme’s position in the metabolic pathway. The right panel indicates, across medusozoan species, the presence of a single transcript (white circle) or more than one transcript (black circle); the protein length (number of residues); and the presence/absence of conserved functional domains, based on NCBI’s Conserved Domain Search. Symbols indicate sequence completeness: *missing N-terminus; – = missing C-terminus; *– = missing both termini. Absent molecules were labeled when the analysis of the top-10 hits in the BLAST search does not allow to confirm the identity of any of the molecules analyzed.

Results revealed PC was consistently recovered across all jellyfish species; however, the CS transcripts were not detected in *Cassiopea xamachana* and *Nanomia bijuga*, and IDH3A was not found in *C. quinquecirrha* and *N. bijuga*, which may reflect incomplete transcript expression profiles rather than the absence of genes.

All the TCA proteins and subunits that compose complexes were identified in the transcriptome of *Stomolophus* sp.2; some were confirmed in the peptide proteomic database. As **Table 2** shows, two isoforms were found for citrate synthase, aconitate hydratase, and malate dehydrogenase; the last two enzymes include cytosolic and mitochondrial isoforms.

**Table 2:** The TCA enzymes from the jellyfish *Stomolophus* sp.2.

Enzyme	E. C. Number	Transcriptome deduced subunits (if applicable)	Number of proteins in proteome	Blast P identity (%) Name of the species and number	
Citrate synthase	2.3.3.1	-	2	89 % ^M^ *R. esculentum* XP_065053736.1	89.87 % ^M^ *R. esculentum* XP_065053997.1
Aconitate hydratase	4.2.1.3	-	1	91.11 % ^C^ *R. esculentum* XP_065063993.1	
			1	92.83 % ^M^ *R. esculentum* XP_065066149.1	
Isocitrate dehydrogenase	1.1.1.41	IDH3G	1	81.27 % ^M^ *R. esculentum* XP_065071342.1	
	1.1.1.42	IDH1	1	90. 82 % ^C^ *R. esculentum* XP_065051222.1	
		IDH2	1	73.36 % ^M^ *P. canaliculata* XP_025089361.1	
2-oxoglutarate dehydrogenase (α-ketoglutarate dehydrogenase)	1.2.4.2	ODOI (E1)	–	–	
	2.3.1.61	ODOI (E2)	1	64.9 % ^M^ *Exaiptasia diaphana* A0A913YGT5 (UniProt)	
	1.8.1.4	DLD (E3)	–	–	
Succinyl-CoA synthetase (succinyl-CoA ligase)	6.2.1.4	SUCA (α)	–	–	
		SUCB1-2 (β)	1	90.14 % ^M^ *R. esculentum* XP_065066710.1	
Succinate dehydrogenase	1.3.5.1	SDHA	–	–	
		SDHB	1	91.21 % ^M^ *R. esculentum* XP_065061130.1	
		SDHC	–	–	
		SDHD (putative)	–	–	
Fumarate hydratase	4.2.1.2	-	-	–	
Malate dehydrogenase	1.1.1.37	-	1	89.70 % ^C^ *R. esculentum* XP_065063466.1	
			1	91.21 % ^M^ *R. esculentum* XP_065061130.1	
*Pyruvate dehydrogenase	1.2.4.1	ODPA y ODPB (E1)	1	88.32 % ^M^ *R. esculentum* XP_065066695.1	
	2.3.1.12	ODP2 (E2)	1	34.4 % ^M^ *Acropora cervicornis* A0AAD9R6L1 (UniProt)	
	1.8.1.4	DLD (E3)	–	–	
*Pyruvate carboxylase	6.4.1.1	-	1	89.83 % ^M^ *R. esculentum* XP_065057010.1	

(**-**) Transcript not detected. E.C. Enzyme Commission number of enzymes. Transcriptome data include all deduced proteins and subunits; abbreviations correspond to gene names or annotation labels in *Stomolophus* sp.2. The number of proteins identified in the proteome of *Stomolophus* sp.2 is indicated, as well as the Blast P identity scores for the protein and species with the highest identity. Gray shaded entries indicate enzymes with two isoforms confirmed in the proteome: (M) indicates mitochondrial, (C) indicates cytosolic.

In addition to the mitochondrial and cytoplasmic malate dehydrogenase isoforms, a third putative paralog with no clear subcellular localization was detected in the *Stomolophus* sp.2 transcriptome. The sequence was initially classified as archaeal-like in PFAM. Then, BLASTp results confirmed its high similarity to uncharacterized oxidoreductases in other cnidarians, suggesting this third sequence may represent a divergent cnidarian paralog whose origin, structure, and function should still be confirmed.

Results show that jellyfish species share a conserved TCA pathway, with differences in the number, types, and locations of enzyme isoforms, consistent with observations in the glycolytic pathway and a putative cnidarian enzyme.

#### 3.1.3 The mitochondrial respiratory chain

Despite the detected limitations inherent to transcriptomic data (including expression-level variability at specific conditions) for *C. sivickisi*, *H. sanjuanensis*, and *Stomolophus* sp.2, representatives of all five canonical mitochondrial complexes were recovered in the analyzed species. **Figure 3** shows all those detected proteins in the five compared jellyfish species. The 13 mitochondrial genome-encoded core subunits were detected across all species including 7 deduced proteins of complex I (ND1–ND6 and ND4L), cytochrome (cyt b), 3 proteins for complex IV (COX1–3), and 2 for complex V (atp6 and atp8).

**Figure 3:**
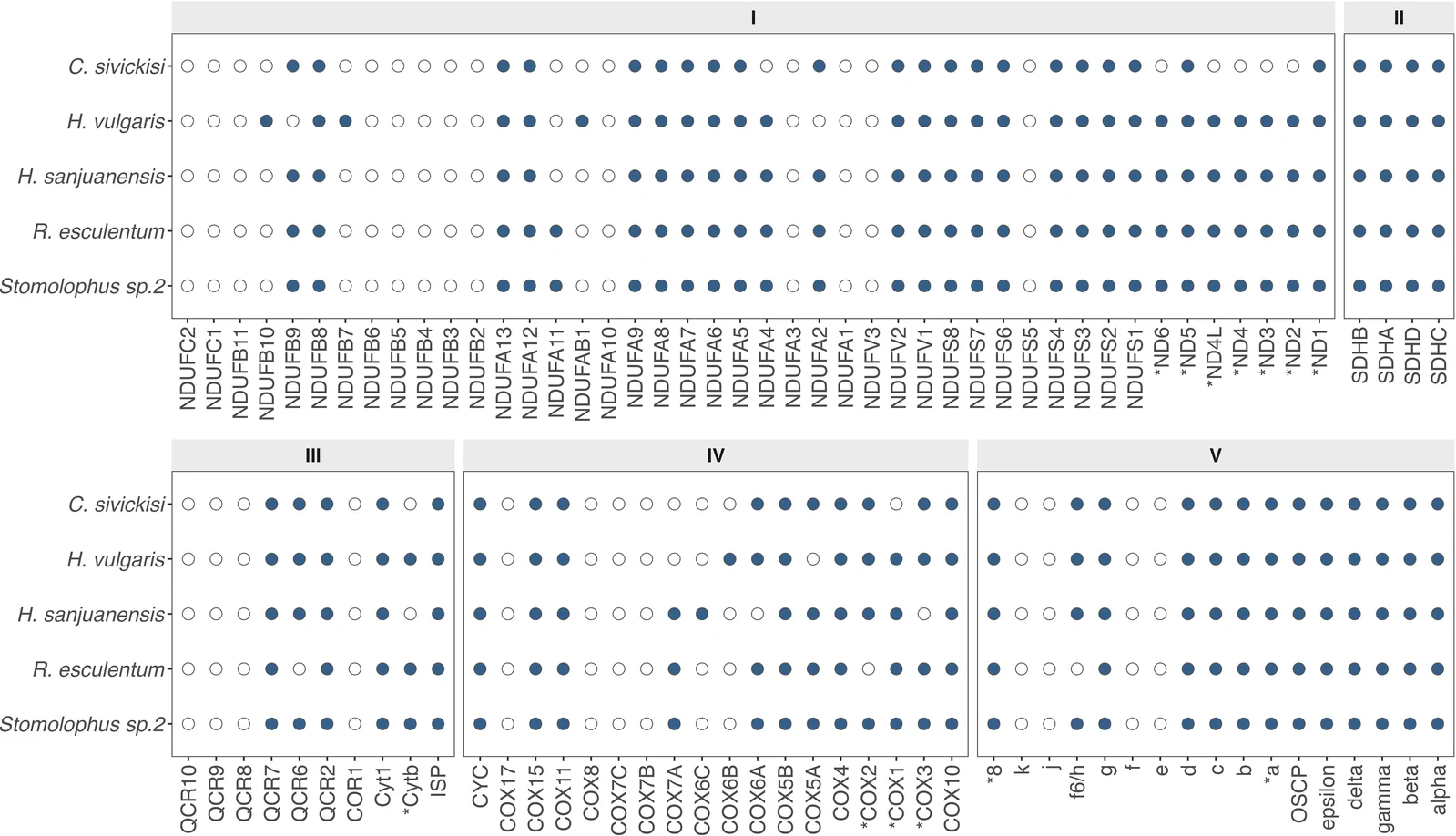
The presence (blue) and absence (white) of deduced proteins or proteome- identified proteins associated with the mitochondrial complexes composing the electron transport chain (complexes I–IV) and ATP synthase (complex V) across five medusozoans species. *C. sivickisi* (Cubozoa), *H. vulgaris* (Hydrozoa), *H. sanjuanensis* (Staurozoa), *R. esculentum*, and *Stomolophus* sp.2 (Scyphozoa). Species names are shown on the y-axis and protein names on the x-axis. Absent molecules were labeled when the analysis of the top-10 hits in the BLAST search does not allow to confirm the identity of any of the molecules analyzed.

All the catalytic, mitochondrial, or nuclear-encoded subunits of each complex were also found across all species. Complex II (succinate dehydrogenase) includes the 4 nuclear-encoded subunits previously identified as part of the TCA. However, several nuclear-encoded subunits were absent in the analyzed databases compared to vertebrate model species, particularly in complexes I, III, IV, and V. These accessory subunits were consistently absent across all analyzed databases, as shown in **Figure 3**. (**Supplementary Table 2** enlists the names of all absent accessory subunits).

To highlight the most conserved and absent elements, a consensus reconstruction of the oxidative phosphorylation pathway was generated. **Figure 4** shows all the proteins detected in at least 3 of the 5 jellyfish species, regardless of data type (transcriptome or genome). The combined results of **Figures 3**, **4** show that Complex I, NADH dehydrogenase includes 28 and tentatively lacks 13 accessory subunits. Complex III, cytochrome c oxidoreductase, includes the three catalytic subunits (cyt b, cytC1, and Rieske ISP) and 3 accessory subunits, but all species lack 4 of the 7 subunits found in vertebrates. The cytochrome c protein (Cyc) was confirmed, and the jellyfish complex IV (cytochrome c oxidase) may include up to 11 subunits, as confirmed in the *Stomolophus* sp.2 transcriptome, out of a total of 17 subunits reported; 4 accessory subunits are missing in all jellyfish species. Finally, complex V (ATP synthase) also lacks four accessory nuclear-encoded subunits in all species. The absence of all these accessory subunits was confirmed in the mitochondrial proteome of *Stomolophus* sp.2.

**Figure 4:**
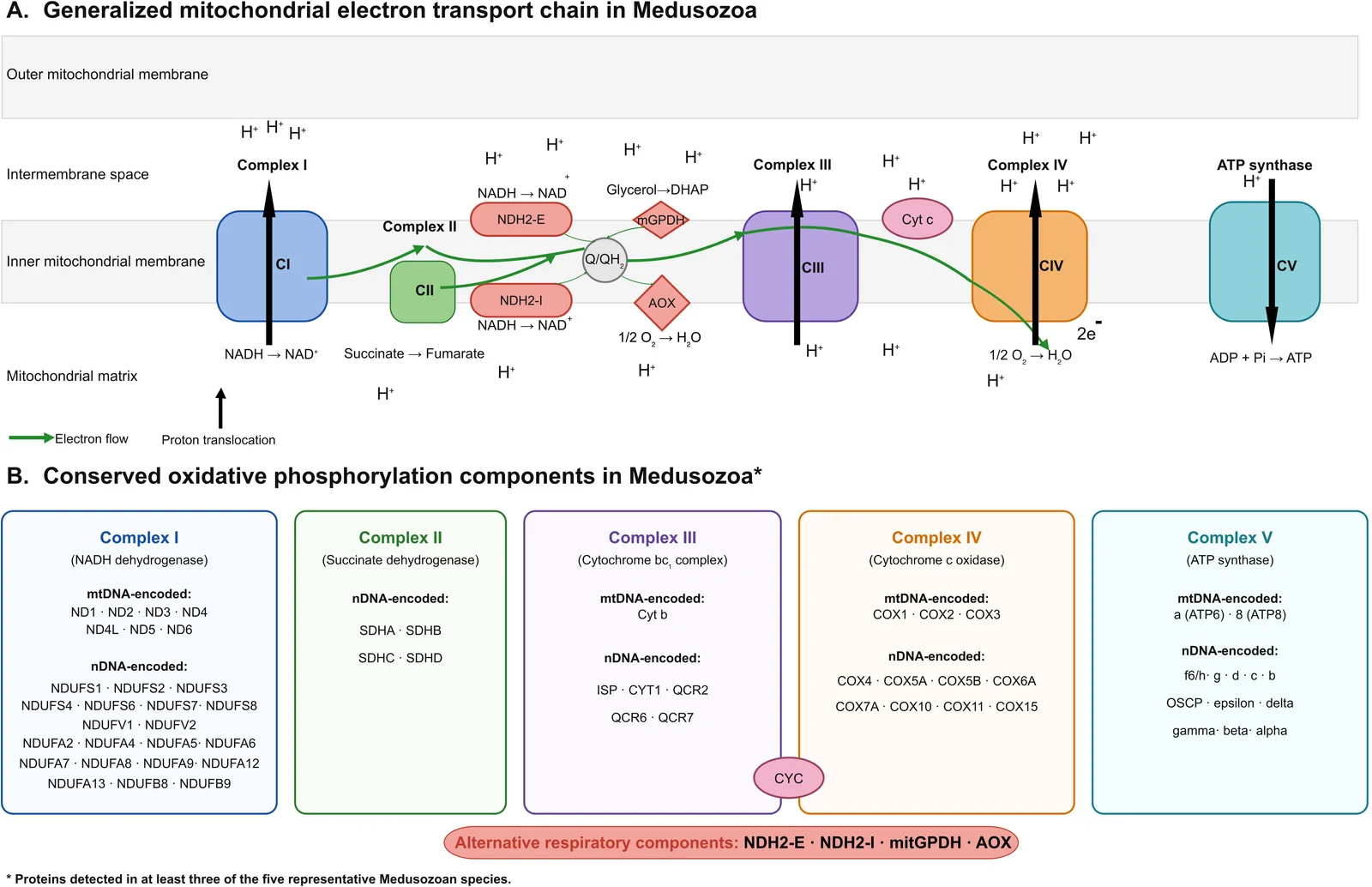
The deduced proteins of medusozoan’s mitochondrial electron transport chain (ETC) and oxidative phosphorylation. **(A)** The pathway template is based and readapted from the *Homo sapiens* KEGG map. Mitochondrial complexes and protein localization are shown in colors, and green arrows indicate the electron flow in the respiratory chain. The four alternative enzymes composing the branched respiratory chain are colored in red. **(B)** Enlisted in color boxes for each mitochondrial complex are the proteins identified in at least three of the five surveyed medusozoan species.

Two Scyphozoan species express an epsilon-like subunit of the F_1_ catalytic domain, which was not detected under its corresponding name but instead as a truncated protein. The deduced protein is shorter in length (45–57 residues) compared to the 63–64 residues in other jellyfish classes, and no peptides were identified in the proteome of *Stomolophus* sp.2 for this protein.

The resulting analysis confirmed the highly conserved nature of catalytic subunits composing the five mitochondrial multimeric complexes; however, major differences were detected in the number of accessory subunits that have been suggested as involved in regulating the enzyme activity and seem to be jellyfish species-specific.

### 3.2 Identification and characterization of specific and novel enzymes

#### 3.2.1 The mitochondrial alternative enzymes of the jellyfish branched respiratory chain

The presence of four alternative enzymes composing the mitochondrial branched respiratory chain of *Stomolophus* sp.2 was analyzed in other jellyfish databases to infer the evolutionarily conserved genes among the jellyfish Medusozoa classes. **Table 3** shows that the alternative oxidase (AOX) was identified in 9 species among the 4 analyzed Medusozoa classes. The jellyfish AOX-deduced protein full-length sequences range from 312 to 336 amino acids in length. Most AOX sequences exhibited the conserved animal-type C-terminal motif N-P-[YF]-X-P-G-[KQE] (McDonald et al., 2009). Notably, both hydrozoan sequences exhibited variations at key positions in this motif, lacking the conserved aromatic and charged residues. The absence of AOX in five species should be interpreted with caution. Four of the five datasets lacking AOX were derived from transcriptomes. Consequently, failure to detect AOX may reflect the absence of gene expression or low transcript abundance.

**Table 3:** Distribution and structural characteristics of two alternative mitochondrial enzymes and the mitochondrial lactate dehydrogenase identified across representatives of Medusozoan.

Class	Species	AOX		mitGPDH		mitD-iLDH	
		Length (aa)	C-terminal motif	Length (aa)	Domain/signature	Length (aa)	Domain/signature
Cubozoa	*Copula sivickisi*	336	NPYGPGE	177	DVLVIGGGATGCGVALESVTRGLKTALVEK	371	GlcD (1-369), FAD-binding PCMH-type (1-127)
	*Chironex yamaguchii*	324	NPFGPGK	725	DVLVIGGGATGCGVALDAATRGLKTALVEK	503	GlcD (44-502), FAD-binding PCMH-type (79-259)
Hydrozoa	*Hydractinia symbiologicarpus y Craterolophus convolvulus*	–	–	588*	–	–	–
	*Hydra vulgaris*	–	–	595	DILVIGGGATGCGVALDACTRGLKTALVEK	–	–
	*Dynamena pumila*	–	–	720	DVLVIGGGATGCGVALDAVTRGLKTALVE	–	–
	*Clytia hemisphaerica*	312	NPLLREH	706	DVLVIGGGATGCGVALDAQTRGLKTCLVEK	–	–
	*Nanomia bijuga*	317	NPLIHHH	106*	–	–	–
Scyphozoa	*Atolla vanhoeffeni*	–	–	267	DVLVIGGGATGCGVALDAATRGLKTALVEK	319	GlcD (1-318), FAD-binding PCMH-type (1-76)
	*Rhopilema esculentum*	323	NPFGPGQ	717	DVLVIGGGATGCGVALDAVTRGLKTALVEK	533	GlcD (58-530), FAD-binding PCMH-type (107-287)
	*Cassiopea xamachana*	317	NPFAPGK	122	DVLVIGGGATGCGVALDAVTRGLRTALIEK	1112	GlcD (647-1109), FAD-binding PCMH-type (686-866)
	*Chrysaora quinquecirrha*	330	NPFAPGQ	723	DVLVIGGGATGTGTALDAATRGLKTALVEK	528	GlcD (63-525), FAD-binding PCMH-type (102-282)
Staurozoa	*Craterolophusconvolvulus*	198	NPFKPGQ	–	–	369	GlcD (1-366), FAD-binding PCMH-type (1-124)
	*Haliclystus sanjuanensis*	133	NPFKPGE	125	DVLIIGGGATGCGVALDAATRGLKTALVE	503	GlcD (42-499), FAD-binding PCMH-type (76-256)

- Not detected. *The identity percentage is over 60% identity with the mitGPDH of *Stomolophus* sp.2 but lacks the conserved signature domain. The domain mappings in medusozoan species. GlcD: FAD oxidoreductase domain; PCMH: bacterial p-cresol methyl hydroxylase family. Numbers in the parenthesis indicate the amino acid positions occupied by each conserved domain within the protein sequence.

As previously mentioned, the mitochondrial isoform of GPDH (mitGPDH) was confirmed in 13 of the 14 jellyfish species (**Table 3**). In *Stomolophus* sp.2, the mitochondrial enzyme shows large differences from the cytosolic variant, including a sequence that is twice as large (SM5). All medusozoans express a mitGAPDH with a confirmed FAD-binding site DVLVIGGGATGCGVALDAVTRGLKTALVE (Brown et al., 1994).

Two additional NADH dehydrogenases were analyzed as part of the four alternative enzymes in the mitochondrial inner membrane. The NDH2-E (external) and NDH2-I (internal), previously reported in the mitochondria of *Stomolophus* sp.2, were also detected in other jellyfish species. Ten of the 13 jellyfish species account for both enzymes, and only one was found in the three remaining species (**Figure 5**). Each enzyme was confirmed by the conserved motif VGGxGxTGxExxxEL, and the binding site for pyridine nucleotides such as NADH or NADPH (Gospodaryov, 2025). All these sequences were confirmed as belonging to jellyfish through their BLAST identity percentages with *Stomolophus* sp.2.

**Figure 5:**
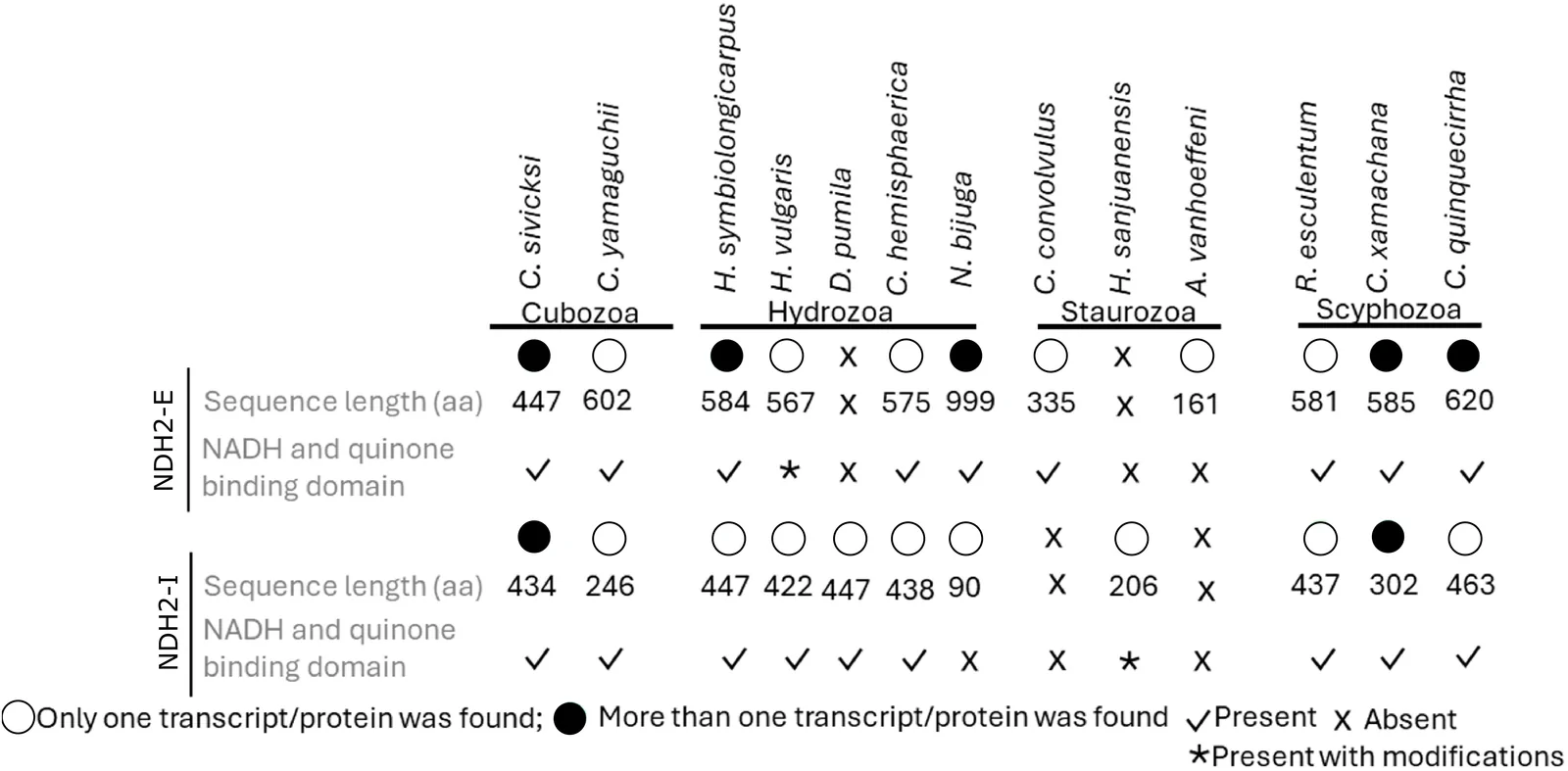
The two-alternative mitochondrial NADH-type2 dehydrogenases (NDH-2) of medusozoans from different taxonomic classes. NDH2-E indicates the external NADH2 dehydrogenase, and NDH2-I indicates the internal NADH2 dehydrogenase. The panel shows, across medusozoan species, the presence of a single transcript (white circle) or more than one transcript (black circle); the protein length (number of residues), and presence/absence of conserved functional domains, based on NCBI’s Conserved Domain Search. Absent molecules were labeled when the analysis of the top-10 hits in the BLAST search does not allow to confirm the identity of any of the molecules analyzed. *Indicates detected subdomains with specific modifications compared to the highly conserved subdomains.

Overall, the results show that all the Medusozoa species analyzed have a branched respiratory chain in their mitochondria, each including at least 3 alternative enzymes, with the number varying by species.

#### 3.2.2 The anaerobic metabolism and jellyfish lactate dehydrogenases

L-lactate dehydrogenase (L-LDH), the enzyme driving anaerobic glycolysis and catalyzing the reversible conversion of pyruvate to lactate in the presence of NADH, was not identified in medusozoans in a search for specific signatures, including NADH-binding sites (Mulkiewicz et al., 2000). The reference transcriptome of *Stomolophus* sp.2 also suggested the absence of the isomer L-LDH transcript but identified one with the characteristic signatures of a D-LDH-like enzyme. The *Stomolophus* sp.2 mitochondrial proteome confirmed the presence of a putative mitochondrial D-iLDH, and the multiple alignment analysis of deduced proteins from homologous transcripts in reference protein databases and phylogenetically close species, such as *Aurelia* sp.1 and *Rhopilema esculentum*, supported the protein identity (SM6).

As **Table 3** shows, except for Hydrozoans, this mitochondrial lactate dehydrogenase (mitD-iLDH) was confirmed in 8 of the 13 analyzed jellyfish species as an NADH^+^-independent LDH, which instead depends on FAD (Andreeßen et al., 2018).

The phylogenetic analysis in **Figure 6** shows that all Medusozoa mitD-iLDH sequences cluster into a single clade, separated from homologous sequences of fish, amphibians, and mammals. Taken together, the results suggest that this novel mitD-iLDH, with specific conserved characteristics among some Medusozoa classes, may indicate the existence of an undescribed mechanism to explain anaerobic jellyfish metabolism.

**Figure 6:**
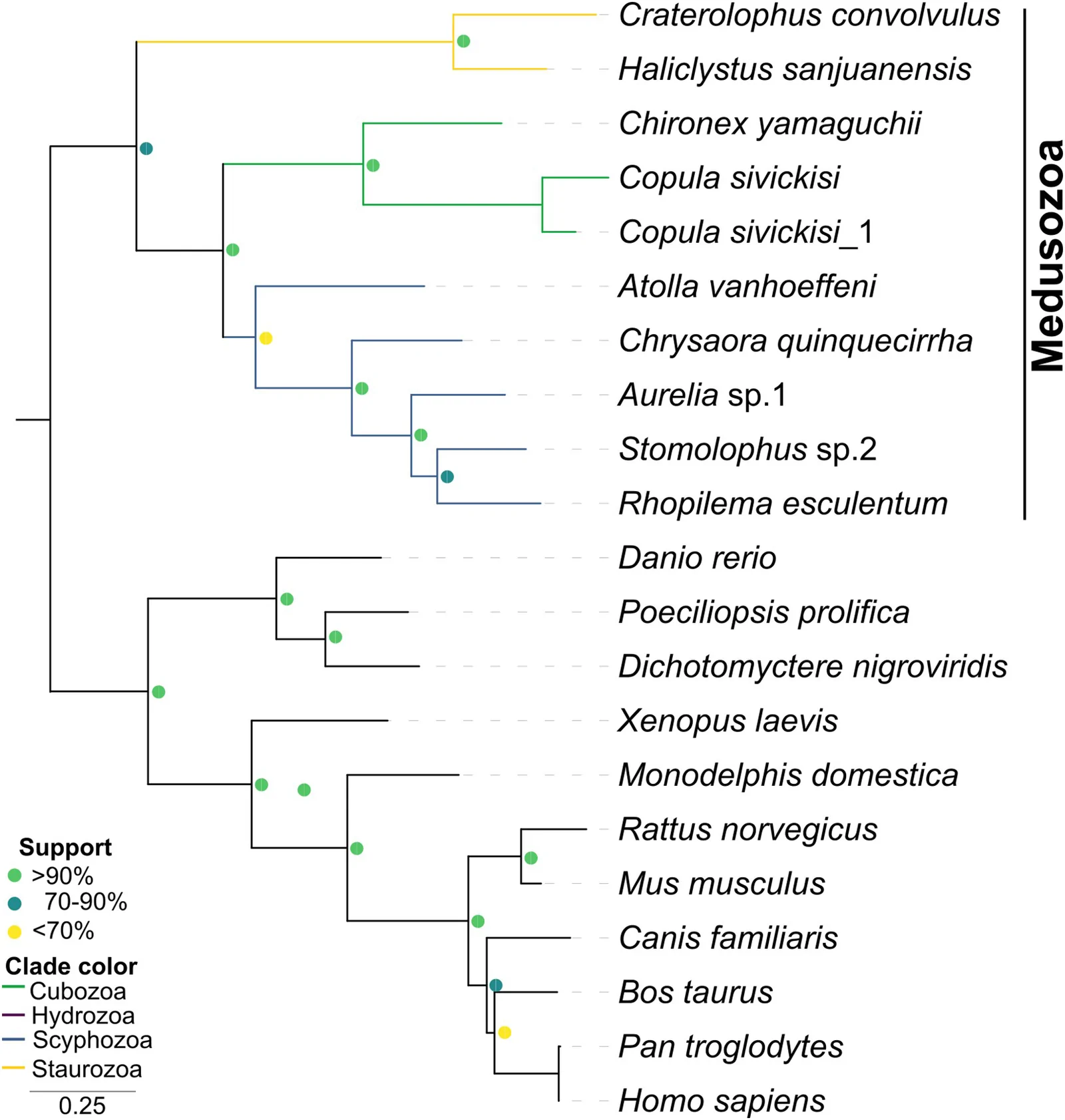
The phylogenetic relationships of mitD-iLDH. IQ-TREE optimal tree (log-likelihood = -18188.04; LG+I+G4) of sequences obtained from medusozoan genomic and transcriptomic datasets. The tree is shown without the rooting taxa (*Kluyveromyces lactis* and *Saccharomyces cerevisiae*).

#### 3.2.3 The jellyfish phosphagen enzymes: creatine- and arginine-kinases

The search for AK in the available datasets did not reveal conclusive evidence of its presence in Medusozoa. Recovered AK sequences corresponded to arthropods, ciliates, and bacteria. Phosphagen kinase sequences previously annotated as AK in the cnidarians *Anthopleura japonica* and *Actinia tenebrosa* did not cluster with confirmed arthropod AKs and did not share the AK-binding motif. Therefore, no confirmed medusozoan AK sequences were recovered from the analyzed datasets.

As **Figure 7** shows, phylogenetic analyses grouped all phosphagen-related sequences from Medusozoa into four major clades, each including representatives from at least three medusozoan classes. This suggests a broad distribution of these phosphagen kinases across the subphylum. Clades 2–4 formed a larger group characterized by conserved phosphagen kinase domains and CK-like binding motifs. Clade 1 occupied a basal position in the phylogeny and, except for *H. vulgaris*, lacked CK-binding motifs but possessed phosphagen-related domains. Clade 4 was distinguished by proteins containing two or three repeated phosphagen-binding regions, whereas most sequences from the remaining clades contained a single phosphagen-binding region. Reference phosphagen kinases from other metazoans formed distinct groups according to their characteristic binding motifs and did not cluster with the medusozoan sequences. All these results support the presence of multiple divergent phosphagen kinases in Medusozoa.

**Figure 7:**
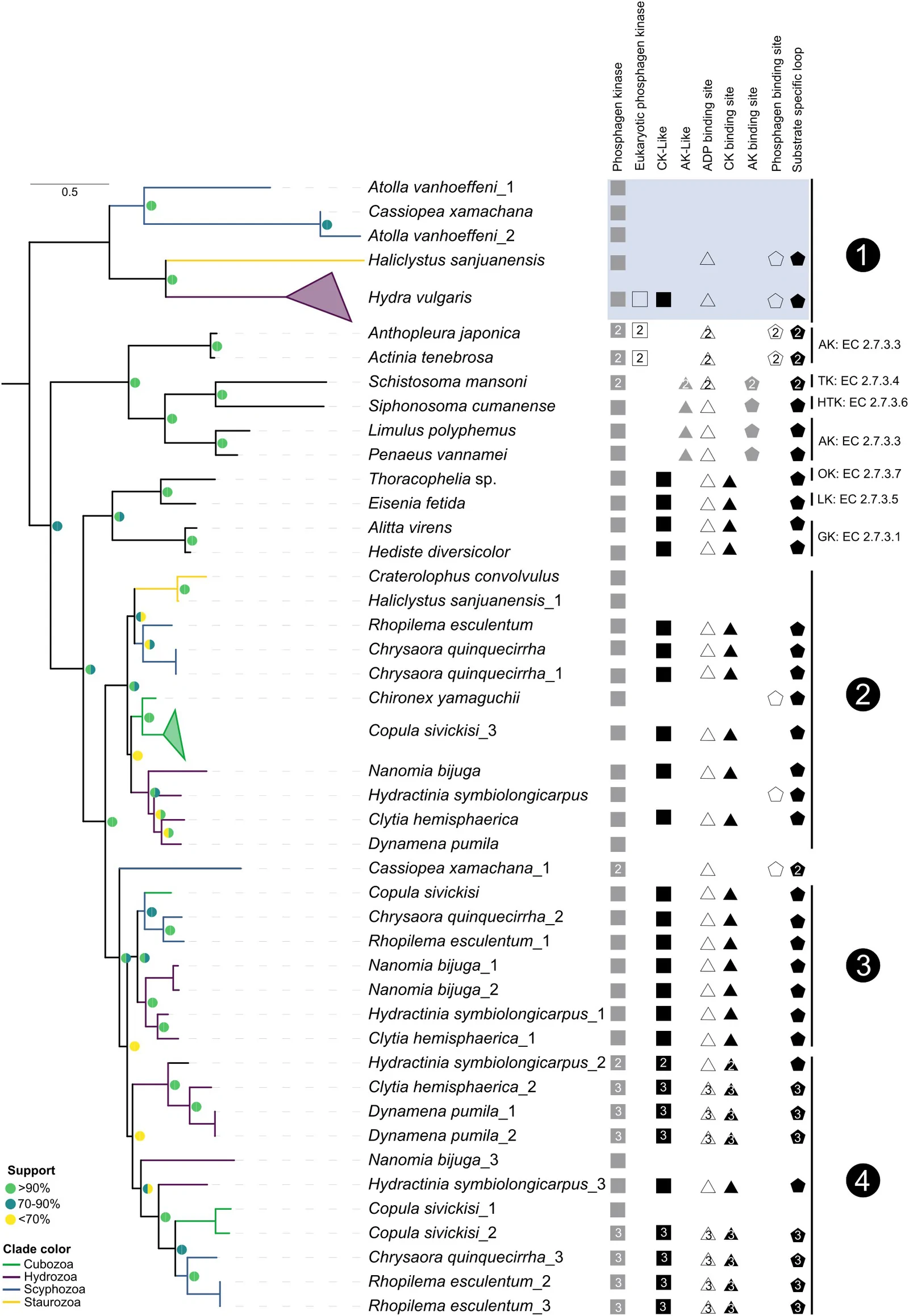
IQ-TREE optimal tree (log likelihood = -29173.82; LG+F+G4) of phosphagen-related sequences obtained from medusozoan genomic and transcriptomic datasets. The four major phosphagen kinase clades are numbered 1-4. The presence of specific protein domains, functional sites and annotations identified during sequence characterization is indicated by symbols next to each terminal. Each symbol represents a specific domain, binding site or functional feature as indicated in the legend. Numbers inside symbols indicate the number of distinct regions containing the corresponding domain, binding site, or annotation. Symbols are absent when the respective domain, site, or annotation is not detected in the sequence. Clade 1 is highlighted in light blue to facilitate visualization of the limited functional annotations in this clade. The tree is shown without the rooting taxon (*Homo sapiens* glycerol kinase).

## 4 Discussion

The ocean is a highly dynamic environment that continuously challenges its inhabitants, which have shown physiological responses and adjustments in their central metabolism to survive. Most animal cell responses are related to the needs of energy transformation and management; thus, molecular adaptations may induce cell reprogramming (MacLean et al., 2023; Dildar et al., 2026).

Glycolysis, as the most evolutionarily conserved pathway in all known animals, is a rapid-response metabolic pathway that compensates for nutrient scarcity, salinity fluctuations, hypoxia, thermal and oxidative stress, among others (Han and Collins, 2012; Ulaje et al., 2019; Wang et al., 2025; Papadopoulos et al., 2025). Previous reports have confirmed that cnidarians may regulate glycolytic and TCA enzymes at the transcriptional/translational levels to cope with hypoxic, acidic or polluted environments (Aljbour et al., 2018; Jesulich et al., 2025); also, jellyfish are known to adjust their metabolic rates, mitochondrial electron transport system, glucose and glycogen contents to meet energy demands driven by thermal stress (Aljbour et al., 2017; Nevarez-Lopez et al., 2020; Gamero-Mora et al., 2025). This study confirms the essentiality and high conservation of glycolytic enzymes in jellyfish; in addition, we suggest that, beyond their role in energetics, important gaps remain regarding their multifunctionality in marine species, which may also be related to the different isoforms detected in this research. Although most glycolytic enzymes have been suggested as potential moonlighting proteins to date (Chen et al., 2026), some, including HK, enolase, and GAPDH, have been extensively studied in this role in non-mammalian animals, such as fish and insects (Gupta and Uversky, 2023). Specifically, GAPDH has been involved in regulating gene transcription, phosphorylating proteins that participate in cell signaling pathways, and contributing to specific responses to stress conditions or diseases (Pancholi, 2001; Gao et al., 2012; Alquraishi et al., 2019; Werelusz et al., 2024). To date, the non-glycolytic role of nuclear GAPDH suggested in marine invertebrates is as part of the apoptotic evasion response after exposure to hypoxia in the whiteleg shrimp *Penaeus vannamei* (Camacho-Jimenez et al., 2023). In jellyfish, the two GAPDH isoforms confirmed in this study may represent molecules with potential as multifunctional proteins; however, for Medusozoa, any hypothetical function deserves further study to explain the biochemical and physiological responses of jellyfish.

The tricarboxylic acid cycle also plays a central role in energetics, as it depends on its thermodynamically favored reactions and its ideal network topology for metabolism, this central player in the organism’s bioenergetics, has been confirmed as a major target of biochemical and physiological adaptations to cellular stressors in the marine environment (Lane, 2022; MacLean et al., 2023; Wang et al., 2024; Mao et al., 2025). Besides some parasites and anaerobic bacteria whose energetic metabolism does not depend on a mitochondrial aerobic respiratory mechanism (Vanacova et al., 2003; Han and Collins, 2012), most organisms depend on the order that the TCA cycle establishes by controlling the production of a series of intermediates and regulating interconnected metabolic pathways. The existence of mitochondrial and cytosolic isoforms of glycolytic and TCA enzymes in the jellyfish repertoire, including iPGM, GAPDH, citrate synthase, aconitase hydratase, and malate dehydrogenase, suggests a benefit-related functional specialization without the need to synthesize new specialized enzymes. Isoforms that differ in structure, regulation, cellular localization, and catalytic properties enhance the jellyfish’s ability to develop specialized physiological responses to environmental challenges (Narahari et al., 2000; Chouchani et al., 2014; Thor et al., 2022).

All the genes or transcripts encoding mitochondrial respiratory chain proteins strongly confirmed the presence of a functional oxidative phosphorylation system across Medusozoa. Although jellyfish may exhibit unusual features, such as a linear mitochondrial genome, they include the 13 conserved proteins encoded in most animals, all of which have been confirmed as subunits of mitochondrial ETC complexes (Kayal et al., 2012).

Except for complex II (succinate dehydrogenase), the absence of some accessory subunits in all the mitochondrial complexes from different jellyfish species is suggested in this study. These results agree with the previous findings of Nevarez-Lopez et al. (2024), who reported lower-molecular-weight active complexes in the isolated mitochondria from *Stomolophus* sp.2 compared with those from bovine heart. In this study, the mitochondrial complex I shows the greatest difference in the number of subunits compared to the enzyme of vertebrates; despite this, medusozoans express all the subunits (7 hydrophilic and 7 hydrophobic) that compose the 14 protein-core of the enzyme and the four ancestral subunits (S6, A9, A12, and S4; Shin et al., 2025). The ATP-synthase complex also differed from that of other vertebrate or invertebrate species; in addition to the four accessory subunits missing in all jellyfish classes, and despite differences in the characteristics of the subunit epsilon homolog, each protein functionality could only be assumed, and requires direct experimental validation.

Although the absence of proteins in jellyfish could reflect expression-level biases in the transcriptomic datasets, their consistent absence across all taxa, including those with genomic data, may suggest a lineage-specific loss or functional replacement, as confirmed in poriferans (Lavrov et al., 2025). However, since most mitochondrial protein complexes have expanded early in eukaryotic evolution through the addition of proteins from the alphaproteobacteriall original complex to the mammals’ larger complexes, the absence of genes in early-branching metazoans such as jellyfish does not necessarily indicate gene loss (Huynen et al., 2013).

As previously evidenced in our group by Nevarez-Lopez et al. (2024), *Stomolophus* sp.2 expresses up to four alternative enzymes as part of a branched mitochondrial respiratory chain. In this species, the confirmed identities and characteristics of alternative enzymes also suggested that they may contribute to the mitochondrial response to thermal stress (Nevarez-Lopez et al., 2024). The widespread distribution of these enzymes in medusozoans, confirmed in this study, supports the hypothesis that this feature is conserved across the subphylum. To our knowledge, there is no evidence of another group of species, including the specific enzymes and characteristics of jellyfish respiratory chains.

Previous evidence has related alternative enzymes in branched respiratory chains to metabolic flexibility, providing various entry and exit points for electrons into the respiratory chain to maintain electron flow and membrane potential, to control ROS production, and to preserve mitochondrial integrity and functionality when the organism faces environmental conditions that may alter the ETC (McDonald and Gospodaryov, 2019). Possessing a branched respiratory chain has been also interpreted as an adaptive strategy that allows mitochondrial electron transfer to follow alternative pathways to the canonical pathway in plants, yeasts, and some animals (Verner et al., 2013; Saari et al., 2019; Juergens et al., 2024).

Previous studies in marine invertebrates have confirmed the participation of an AOX as an alternative to the reaction catalyzed by complex IV and as the adaptive physiological response of bivalves and crustaceans to hypoxia and reoxygenation (Yusseppone et al., 2018; Rodriguez-Armenta et al., 2018). In addition, an active mitGPDH was confirmed in the branched respiratory chain of *Artemia* nauplii and suggested as a mitochondrial uncoupling mechanism (Rodriguez-Armenta et al., 2018). The existence of four alternative enzymes in a branched ETC, including an external NDH2-E (tentatively located in the intermembrane space) and an internal NDH2-I (tentatively located on the inner side of the mitochondrial inner membrane), is rarely found in animals.

To date, the specific functional roles of alternative enzymes remain unclear in most marine invertebrates. Still, common features have been described for species whose mitochondria express alternative enzymes, including sessile species or a sessile phase during their life cycle, and tolerance to environmental stress such as hypoxia, hypersalinity, dehydration, and extreme heat or cold seawater (McDonald and Gospodaryov, 2019). Although the occurrence of AOX or NDH2 proteins in animals is rare, the co-expression of both proteins in a branched respiratory chain is commonly associated with marine invertebrates repeatedly exposed to hypoxic and reoxygenated environments (McDonald and Gospodaryov, 2019). These characteristics are consistent with some cnidarians, especially medusozoans. However, the function and specific role of each enzyme in the mitochondrial machinery responses to environmental changes should be carefully studied and experimentally confirmed.

The basis of jellyfish anaerobic metabolism was also explored in this research. Although the lactate pathway is the dominant via utilized by animals to generate ATP during hypoxia/anoxia, and despite previous reports suggesting the involvement of an L-LDH in the anaerobic glycolytic pathway of jellyfish, and the lactate detection in jellyfish cells as the result of exposure to hypoxia (Wang et al., 2017; Nevarez-Lopez et al., 2020), the search for L-LDH homologs in medusozoans did not yield a confirmed sequence of any gene or transcript encoding the enzyme in all the analyzed species.

In nature, animals lacking L-LDH are rare; one example is the recently reported scallop *Chlamys farreri*, whose anaerobic metabolism relies on alternative routes such as the glucose-succinate pathway (Bao et al., 2024). Thus, the first question is the identity of the enzyme(s) and pathways that drive anaerobic metabolism in jellyfish. Previous studies have confirmed the occurrence of up to three additional alternative anaerobic pathways in marine invertebrates for re-oxidizing NADH under anaerobic conditions, including the glucose-succinate, aspartate-succinate, and glucose-opine production pathways (Harcet et al., 2013; Cox and Gillis, 2020).

Previous evidence in early branching metazoans suggests that poriferans (sponges) produce a specific sponge-type opine dehydrogenase which belongs to the ornithine cyclodeaminase

(OCD)/mu-crystallin family (Harcet et al., 2013). However, there is still no evidence of dehydrogenase or oxidoreductase enzymes participating in the catalytic reactions of any of these alternative anaerobic pathways in the jellyfish databases, which opens an opportunity to deepen our understanding of medusozoans.

Another feasible explanation for the jellyfish anaerobic metabolism may involve an LDH isomer. In 2026, Pasos-Ramirez (Pasos-Ramirez, 2026) reported for the first time in cnidarians a mitD-iLDH from the jellyfish *Stomolophus* sp.2 (GenBank acc.no PV915641.1); our results now confirmed the presence of the enzyme in most Medusozoa classes. Few studies of mitD-iLDH confirm its location in the mitochondrial matrix, as detected in *Mus musculus* (de Bari et al., 2002, 2019; Jin et al., 2023). The enzyme is suggested to catalyze the conversion of D-lactate to pyruvate, which is then metabolized into acetyl coenzyme A to enter the Krebs cycle. The FADH_2_ molecules resulting from mitD-iLDH activity may serve as electron donors for the reduction of ubiquinone to ubiquinol, thereby feeding the ETC; then FADH_2_ is oxidized again to FAD^+^ and reused in D-lactate metabolism (de Bari et al., 2002; 2019). The energetic advantage of an active mitD-iLDH suggests that mitochondrial lactate can be converted to pyruvate in the mitochondrial matrix, thereby feeding the ETC rather than being discarded (de Bari et al., 2002; 2019). Future research still requires experimental evidence to confirm the activity and role of jellyfish mitD-iLDH in mitochondrial isolates from jellyfish exposed to environmental challenges.

Finally, the lactate levels previously reported in the cytosol of jellyfish bell cells (Nevarez-Lopez et al., 2020), could be partially explained as the final product of a detoxification process that controls methylglyoxal levels produced during hypoxia/anoxia stress (Lai et al., 2022). Methylglyoxal is a toxic byproduct produced from glycolysis, which is metabolized by the glyoxalase pathway into D-lactate, which then enters the mitochondrial matrix through a monocarboxylate transporter (de Bari et al., 2019; Scheckhuber, 2019).

Among the alternative jellyfish energy sources described in this study, phosphagens have recently emerged as a complex system that rapidly releases additional energy. Arginine phosphate and arginine kinase represent the phosphagen system commonly found among marine invertebrates. However, the results did not provide convincing evidence for the presence of AK in Medusozoa. This finding is notable since AK has been reported in Anthozoa, the sister group of Medusozoa. There is evidence of AKs with up to three phosphagen kinase domains in anthozoans (Uda et al., 2012; Matsuo et al., 2017); however, the anthozoan sequences included in the phylogenetic analysis (GenBank: XP_031557190.1 and UniProt: O15992) did not cluster with confirmed arthropod AKs and lacked the characteristic AK-binding motif. All these observations suggest that canonical AK may be absent or highly divergent in Medusozoa. Additional genomic resources and broader taxonomic sampling will be necessary to determine whether Medusozoa truly lacks AK or possesses highly modified phosphagen kinases that remain unrecognized by the homology-based approaches here used.

Instead of an AK, the CK presence was confirmed in Medusozoa and phylogenetic analyses revealed a basal cluster that lacks CK-like binding sites in four of the five species included in that clade. The absence of CK-like binding sites in several sequences may reflect either genuine sequence divergence or incomplete assemblies. Similar diversification of phosphagen kinases has been described in annelids (Suzuki et al., 2009), including opheline kinase (OK), lombricine kinase (LK), and glycocyamine kinase (GK) (Yano et al., 2022). Results in **Figure 7** showed that the OK, LK, and GK sequences also possess CK-like binding sites, indicating that the presence of these motifs is not restricted to conventional CKs. These observations suggest that the recovered medusozoan proteins may represent distinct phosphagen kinase lineages, although the possibility that some correspond to CK isoforms similar to those reported in the anthozoan *Nematostella vectensis* (Uda et al., 2012) cannot be excluded. Functional and biochemical characterization of these enzymes will be required to determine their substrate specificity and establish whether these clades correspond to previously unrecognized phosphagen kinases in Medusozoa or already described isoforms.

The presence of four distinct phosphagen kinase clades raises the possibility that this diversity resulted from gene duplication followed by subsequent divergence within the medusozoan lineage. However, the present phylogenetic analysis does not provide sufficient evidence to distinguish this scenario from the presence of previously unrecognized phosphagen kinase lineages that diverged early in medusozoan evolution. Broader genomic sampling and analyses of gene organization and orthologous relationships will be necessary to distinguish between these hypotheses. Further analyses will also be needed to investigate the domain expansion observed in some proteins, including the presence of multiple phosphagen kinase regions in Clade 4.

One limitation in this effort to reconstruct medusozoan bioenergetics is the still scarce availability of databases for these species. The interpretation of the presence or absence of specific genes or transcripts across different tissues, developmental stages, and experimental conditions should account for the heterogeneity of the available datasets. Some analyses also relied on predicted proteins from genome assemblies, which may vary in quality, sequence coverage, and completeness. Failure to detect a protein should not necessarily be interpreted as evidence of its biological absence. However, in this study, consistent patterns recovered across multiple species, despite the aforementioned limitations, may provide a robust molecular framework for jellyfish bioenergetics. This reconstructed basis provides a foundation for future physiological and biochemical studies of how Medusozoa respond to and adapt to changing environmental conditions.

The integrated findings in this study suggest that jellyfish species share specialized strategies for energy transformation, including the synthesis of specific molecules that sustain and regulate mitochondrial energy metabolism, as previously suggested by Gamero-Mora and Muhlia-Almazan (2025). Overall, the results of this study confirm this ability and suggest that jellyfish incorporate into their repertoire molecules that make up energy pathways, conserved regulatory enzymes, as well as specific isoforms or species-specific molecules, allowing them to transform enough energy to face environmental challenges, maintain the diversity of forms involved in their life cycle, and bloom.

## Data Availability

The datasets presented in this study can be found in online repositories. The names of the repository/repositories and accession number(s) can be found in the article/**Supplementary Material**.
