# Supplementary material for: Comparative analysis of bioenergetic pathways in Medusozoa based on integrated genomic, transcriptomic and proteomic data
Source: Front Physiol. 2026 Oct 5;17:1932200. doi: 10.3389/fphys.2026.1932200 (PMC13679545; doi:10.3389/fphys.2026.1932200)
Supplement: Supplementary file 1 [file DataSheet1.docx]

**Supplementary Material**

**Electronic supplementary material index:**

| Supplementary Material 1  SM1 | The analytical workflow including the bioinformatic pipeline used to reconstruct the bioenergetic metabolic pathways of Medusozoa |
| --- | --- |
| Supplementary Material 2 SM2 | Multiple alignment of Medusozoan hexokinase protein sequences. |
| Supplementary Material 3  SM3 | Multiple alignment of Medusozoan phosphofructokinase (PFK) protein sequences. |
| Supplementary Material 4  SM4 | Multiple alignment of Medusozoan pyruvate kinase (PK) protein sequences. |
| Supplementary Material 5  SM5 | The two GAPDH isoforms of the jellyfish *Stomolophus* sp.2. |
| Supplementary Material 6  SM6 | Multiple alignment of animal mitD-iLDH protein sequences. |
| Supplementary Table 1  ST1 | Reference protein sequences used for the phylogenetic  analyses shown in Figures 6 and 7. |
| Supplementary Table 2  ST2 | The nuclear-encoded subunits from mitochondrial complexes not detected across the five representative Medusozoan datasets including the *C. sivickisi* transcriptome, *H. vulgaris* genome, *H. sanjuanensis* transcriptome, *R. esculentum* genome, and the *Stomolophus* sp.2 transcriptome. |

**SM1. The analytical workflow including the bioinformatic pipeline used to reconstruct the bioenergetic metabolic pathways of Medusozoa.**

```
Nanomia bijuga                     ------------------------------------------------------------	0
Hydra vulgaris                     ------------------------------------------------------------	0
Copula sivickisi                   ------------------------------------------------------------	0
Chironex yamaguchii                ------------------------------------------------------------	0
Rhopilema esculentum               MEIGMHDVSNMETECSSCEDAMKRDRHDSCLCEDCLMQSINFFGQQESVLERRFKRRSVT	60
Chrysaora quinquecirrha            ------------------------------------------------------------	0
Cassiopea xamachana                ------------------------------------------------------------	0
Haliclystus sanjuanensis           ------------------------------------------------------------	0
Craterolophus convolvulus          ------------------------------------------------------------	0
Hydractinia symbiolongicarpus      ------------------------------------------------------------	0
Dynamena pumila                    ------------------------------------------------------------	0
Clytia hemisphaerica               ------------------------------------------------------------	0


Nanomia bijuga                     ------------------------------------------------------------	0
Hydra vulgaris                     ------------------------------------------------------------	0
Copula sivickisi                   ----------------------------------------------------------MD	2
Chironex yamaguchii                ----------------------------------------------------------MH	2
Rhopilema esculentum               SMDDIHQKIIKEPKMDMDIVSP---VWCPSSTVAEQPMMLGPSRNMYSDVLSRYREGEMF	117
Chrysaora quinquecirrha            ----------------------------------------------------------MF	2
Cassiopea xamachana                -----------MSILEYRSLVPLPSEKCSNA-VGKH-VAL--KNVNEDHTDSKILEGEMF	45
Haliclystus sanjuanensis           ------------------------------------------------------------	0
Craterolophus convolvulus          ------------------------------------------------------------	0
Hydractinia symbiolongicarpus      ------------------------------------------------------------	0
Dynamena pumila                    ------------------------------------------------------------	0
Clytia hemisphaerica               ------------------------------------------------------------	0


Nanomia bijuga                     ---------------------KVSEYLKTLELPNGSLELLIKRLHENFNIGLTPDDTPGK	39
Hydra vulgaris                     ---MYLSQLRRADLPGRWEKEELEKLFNLFELQRPELEKLITVFQKDIADGLSKTSH---	54
Copula sivickisi                   SVIPHQISSPAGAAPQLWEKEKVKQVIDPFLLSKEALENVISAMNEDFDHGLSNDPSL-Q	61
Chironex yamaguchii                SVIHPHLTSTNKGAPELWEQERVRAVLDGFLLSKATLELVIQAMDEDFTNGLSSDPEM-N	61
Rhopilema esculentum               SMIPYVVDLSKKLIPGLWEKEKLKEVLNKFILTKDTLEQVIAAMNEDFDNGLSNDPER-S	176
Chrysaora quinquecirrha            SMIPYVVDLSKKAVPGLWEKEKLKDVLNKFILTKETLEKVMSAMNEDFDNGLSCSPER-N	61
Cassiopea xamachana                SMIPYVVDLSKRLVPGLWEKEKLKEILNKFILSKETLEKVMYAMNEDFENGLSCDPER-S	104
Haliclystus sanjuanensis           ------------------------------------------------------------	0
Craterolophus convolvulus          ------------------------------------------------------------	0
Hydractinia symbiolongicarpus      ------MQSLRQGVPGEWEKERVNDVLAQFVVSRETLESVMLKFNEDFTLGLSSEQTK-R	53
Dynamena pumila                    -----MYPPAHQEIPGTWERGKVKALLDQFKVSRESLEQMILEMDKDFNKGLSDSSSV-R	54
Clytia hemisphaerica               -----MYPATHQCAPGAWEKEKVKTLLKKFEFPRESAEELISKMNEDFSYGLKNGGD---	52


                                                               * *
Nanomia bijuga                     ECGTIKMLPTYVRDIPQGTKTEEGDYFALDLGGTNFRVLHVQIQDGKITSSHDTFAVSDE	99
Hydra vulgaris                     DVARCKMLLTYVQEKPNGT--ENGNFFALDLGGSNFRVLLVQINDGIIKKLQRTYPVSKD	112
Copula sivickisi                   DKSPIKMLLTYIRSKPDGK- EEGKFFALDLGGTNFRVLLVNIHAGQITLKHQKYPVDDS	119
Chironex yamaguchii                RLSPIKMFPTYIRSKPDGS--EEGNFFALDLGGTNFRVLLVRIDHGQISMQHQTFPMDDS	119
Rhopilema esculentum               HKSPIKMLVTYVRSTPNGS--EKGDFYALDLGGTNFRVLLVEIHDKKISMSHKQYAMDDA	234
Chrysaora quinquecirrha            HRSPIKMLVTYVRSTPNGS--EKGDFYALDLGGTNFRVLLVKIHDKKIDMTSKQYAMDDS	119
Cassiopea xamachana                HKSPIKMLVTYVRSTPNGT--EEGDFYALDLGGTNFRVLLVEIHDKKIAMSHEQYAMDDS	162
Haliclystus sanjuanensis           ------------------------------------------------------------	0
Craterolophus convolvulus          ------------------------------------------------------------	0
Hydractinia symbiolongicarpus      DQSPVKMLPTYVRSIPDGT--EEGDYFALDLGGTNFRVLLVSIKNNQISMSHKTYPMGSA	111
Dynamena pumila                    SESPIKMIPTFVRAVPDGS--EEGDFFALDLGGTNFRVLLVKIHCGNAKMSHSTYPIAPD	112
Clytia hemisphaerica               YHSPVKMLLTYVHGLPDGT--ETGDYFALDLGGTNFRVLLVKLNDGNVSMSHETFPISQE	110


                                           *   *   *  *                * * *		*
Nanomia bijuga                     LMKGNGDDLFNFVAEKLAGFAVRKLSCEKITITALAFTFSFPVDQKSLYSGSLIKWTKGF	159
Hydra vulgaris                     IMNSSRDALFGYIAECLASFVKETLGENHKRIEVIGFTFSFPVKQTSLTKGTLIKWTKGY	172
Copula sivickisi                   KMKGSADDLFGYIAESLAKFAKENG-VTDGKLKSVGFTFSFPVKQSSLFSGSLVKWTKGY	178
Chironex yamaguchii                KMKGTAEVLFGYIAECLAKFSREHG-VTDGKLTFVGFTFSFPVIQTSLFSGKLLKWTKGY	178
Rhopilema esculentum               LMKGSGSNLFAYIAECLAKFSNEHK-V--NKLCSVGFTFSFPIDQKSLTSGNLIKWTKGY	291
Chrysaora quinquecirrha            LMKGTASKLFGYIADCLAKFAGEHQ-V--NKLCSVGFTFSFPIHQTSLTSGNLIKWTKGY	176
Cassiopea xamachana                LMKGTGSKLFGYIAECLANFANHHK-V--DTLCSVGFTFSFPIDQKSLTSGNLIKWTKGY	219
Haliclystus sanjuanensis           ------------------------------------------------------------	0
Craterolophus convolvulus          ------------------------------------------------------------	0
Hydractinia symbiolongicarpus      IMEGTAENLFGYIAQCLAEFGKEKLGEDLKKIAAVGFTFSFPVEQTSLTSGNLIKWTKGY	171
Dynamena pumila                    MMTGSADMLFGYIAECLAKFAKEQLGEGTKKIATVGFTFSFPVDQTSLVSGKLIRWTKGY	172
Clytia hemisphaerica               IMKGDADGLFGYIAECLAKFAKLKLGQETRKLGTVGFTFSFVRQESLTCGSLIKWTKGFY	170


				 *   *
Nanomia bijuga                     CASGVEGEDVVGLLNKAFNKREVSF-----------------------------------	184
Hydra vulgaris                     NISGVVDHDIIQLLSEAIEKQQEISVGKIILLNDTTGVMMCGAYDDPEVEIGLILGTGSN	232
Copula sivickisi                   TTSGVEGEDVVKLLHNAIRKRKEIEVERIALLNDTTGTMVSCAFEDQNVEIGLILGTGTN	238
Chironex yamaguchii                TASGVEKEDIVKLLHEAIERRNEIQVEKIALLNDTTGTMVSCAFDDREVEIGLILGTGTN	238
Rhopilema esculentum               SADGVEGKDVVQLLHNELDKRDDLKVHKVALVNDTTGTMVACAFDDPDVEIGLILGTGTN	351
Chrysaora quinquecirrha            SADGVEGKDVVRLLHDCIDQRDDFSVNKIALVNDTTGTMVSCAFDDPDVAIGLILGTGTN	236
Cassiopea xamachana                SADGVEGKDIVKLLHDEVDKRDDLKVNKIALVNDTTGTMVACAFDDPDVAIGLILGTGTN	279
Haliclystus sanjuanensis           ------------------------------------------------------------	0
Craterolophus convolvulus          ----------------EIVKRKDLNVGKIALVNDTTGTMVSCAFDDPNVRIGLILGTGTN	44
Hydractinia symbiolongicarpus      NVAGVEGNDIIKLLTDAIERRKEIEVEQIALLNDTTGVMMSCAFYDQAVAIGLILGTGTN	231
Dynamena pumila                    DVKGAEGNDIVKLLKAAIAKRNEIEVEEIALLNDTTGVMMSCAYGDPTVAIGLILGTGTN	232
Clytia hemisphaerica               DVDDVEDHDVVALLQQAIEKRNEIEVEQIALLNDTTGVMMSCAYNDPDVMVGMILGTGSN	230


Nanomia bijuga                     ------------------------------------------------------------	184
Hydra vulgaris                     ACYMEKLENIEKFEEVL--SGPQQFIVNMEWGAFGERDQLAFVATKFDLMVDEKSIHPGQ	290
Copula sivickisi                   ACYMESLDRVQKWNGDS--NEPRQVIVNTEWGAFGEKGELNSVKTFADEAVDSSSLYPGQ	296
Chironex yamaguchii                ACYMESLDMVKKWDGEL--DGPKQVIINTEWGAFGEKKELDFVKTDFDRIVDGGSLYPGQ	296
Rhopilema esculentum               ACYMERTSNIPKWDGDD--GPPKQVIINTEWGAFGEKGELDFVKTDYDIKVDQESLYPGE	409
Chrysaora quinquecirrha            ACYMEKLENIPKWDGDQ--GNPKQVIINTEWGAFGEKGELDFVKTEYDIKVDKNSLYPGE	294
Cassiopea xamachana                ACYMEKTSNIPKWDGDE--GPPKQVIINTEWGAFGERGEIDFVKTEYDIRVDQESLYPGE	337
Haliclystus sanjuanensis           ---------------------------NTEWGAFGEKGELDFVKSKFDDIVDRESLYPGQ	33
Craterolophus convolvulus          ACYMERLDRVGKWDGDC--MEPKEVIINTEWGAFGERGETFSVATKFDELVNRESLYPGQ	102
Hydractinia symbiolongicarpus      ACYMEDISSVKKWDGDL--NEPKQVIVNTEWGAFGEKGELRCVATQFDDIVDQESLYPGQ	289
Dynamena pumila                    ACYIESLERVRKWKQIEGDSAPDQVIINTEWGAFGDKGEINDLRTKYDLVLDDKSLYPGK	292
Clytia hemisphaerica               ACYMEKLEYVGKWFHPQ--SPYKECIINTEWGAYGDMDELQSVSTSYDKIVDEESLYPGK	288


Nanomia bijuga                     ------------------------------------------------------------	184
Hydra vulgaris                     QIFEKMISGMYLGELVRLCIVELIQKCILFHGVISKEIQNMDAISSEDVSVFCGGVASEA	350
Copula sivickisi                   QIYEKMISGMYLGEIARHVLVQLVKEKILFNGHLTDTLAKEGSFLSSYVSDIASDNAEKV	356
Chironex yamaguchii                QIYEKMISGMYLGEVARQVLLQLIKDNLLFDGHLTPAITKEGSFETRYVSDICSDDAAKI	356
Rhopilema esculentum               QVYEKMISGMYLGEVARQVLVELINEKVIFNGLVTPLLQKTGSFETRFLSDIASDEAKKI	469
Chrysaora quinquecirrha            QVYEKMISGMYLGEVARQVLLDLVKEGVVFQGVVTPQLEKQGSFETRFLSDIASDDAKTI	354
Cassiopea xamachana                QVYEKMISGMYLGEVARQVLVELVNEKVLFNGVVTPMLQKIGSFETRFLSDIASDDAKKI	397
Haliclystus sanjuanensis           QNYEKMISGMYLGEIARQAILELIREGVLFDSRVSDKLQKPGSFESRYLSDVASDDAKKV	93
Craterolophus convolvulus          QIFEKMISGMYLGEIARQAILELIRQGVLFDSRVSDELQKTGSFESRFLSDVASGDATDI	162
Hydractinia symbiolongicarpus      QLYEKMISGMYLGEIVRQCLLHLIKMFVLFRGKVSSQLAVQNSFLTMYVSDFCSGEVKNI	349
Dynamena pumila                    QVFEKMISGMYLGEIVRQCLIGLISDYTLFRGRISEQLAQKNSFTTIYVSEFCSGRAKDV	352
Clytia hemisphaerica               QLFEKMISGMYLGEIVRQCILSLIKDFVLFRGYISPQLSKKDSFTTIYVSEFCSGDAMKC	348


Nanomia bijuga                     ------------------------------------------------------------	184
Hydra vulgaris                     IAILCNLGYKDALVEEILIIQDLCFAISRRSATLTSAGLAALIRRMGKKRVSVAIDGSLF	410
Copula sivickisi                   LKIVQTLGYE-PTLRDLQRVKDVCDAVSIRAARLAAAGLAAIIRRIQKERITVAVDGSLF	415
Chironex yamaguchii                LKILQGFGYE-PALRDLQRVKDVCDAVSIRAARLAAAGLATIIRRIKKKAVTVAVDGSLF	415
Rhopilema esculentum               LEVLEHLGYK-PNLNELQVVKDVCDMISMRSARLAAAGLATIIRRLDKKKLTVAVDGSLF	528
Chrysaora quinquecirrha            LGVLEQLGYN-PTLKELQIIKDVCDMVSMRSARLAAAGLATIIQRLDKNKLTVAVDGSLF	413
Cassiopea xamachana                LDVLEHLGYK-PSLNELQIVKDVCDMVSMRSARLAAAGLATIIKRLGRNKLTVAVDGTLF	456
Haliclystus sanjuanensis           ITILEDLGYENPTLEACQIVKDVCDAVSFRSARLAAAGLATIVKRIGDKKLTVAVDGSLF	153
Craterolophus convolvulus          LSVLSQVGYTRPSLEECQAIKDVCDAVAYRSARLAAAGLATIVKRIDEKKLTVAVDGSLF	222
Hydractinia symbiolongicarpus      HKILKLLGYEQATLFECQVIRDVCNVVSLRAARLAAAGLTTIVRRLDKNSLTVAVDGSLF	409
Dynamena pumila                    IGILEKLGYKNATLEDCCVVQEICLGVSHRAARLSAAGLTAIIRRLKKDSLSVAIDGTLY	412
Clytia hemisphaerica               VNILHQLGYEAATIEDCHVMQDICIAVSLRAARLAAAGLTCILRRLKRSSVSVAMDGSLY	408


Nanomia bijuga                     -----------------------------------------------------		184
Hydra vulgaris                     KNHPTYKMFMYDALRMLLPTVTVKLVLAEDGSGKGAALVAAVVQRLDQPSN--		461
Copula sivickisi                   KYHPKFKSYMEVALENLVPQVKVTLTLSEDGSGRGAGLAAAVAERA-------		461
Chironex yamaguchii                KKHPKFKDYMALALENLVPECKVKLRLSEDGSGRGAALAAAVARRLSMKGSA-		467
Rhopilema esculentum               KKHPKFKSYMAETLLELVPDXKVKLMLSEDGSGKGAALVAAVASRLEAENQ--		579
Chrysaora quinquecirrha            KKHPKFKGYMEEALTELVPHCRVKLMLSEDGSGRGAALVAAVASRLDSQV---		463
Cassiopea xamachana                KKHPKFKGYMEETLVELVPECTVKLMLSEDGSGKGAALVAAVASRLESEMN--		507
Haliclystus sanjuanensis           KKHPKFKGYMATTLRELVPDHTIKLMLSEDGSGRGAGLVAAVSSRLESK----		202
Craterolophus convolvulus          KKHPHFKSYMASTLRELVPQYAVKLMLSEDGSGRGAGLVAAVSSRLDQAST--		273
Hydractinia symbiolongicarpus      KKHPTFKRFMYEALDELLPTTQVKLRLAEDGSGQGAALVAAVANRLSKK----		458
Dynamena pumila                    KKHPTFKRFMHQALEELVPTTVVHLQLAEDGSGRGAAVVAAVADRLSRSNAEA		465
Clytia hemisphaerica               KKHPTFKRFMHQALEEFLPTTQVNLRLAEDGSGRGAAVVAAVAQRLAKTALVD		461
```

**SM2. Multiple alignment of Medusozoan hexokinase protein sequences.**

The conserved G residues are indicated in black, shaded residues are conserved or highly similar to the mammalian HK primary structure, and asterisks indicate the conserved hydrophobic residues that form the HK channel (Kuser et al., 2000). Accession numbers are not provided since sequences resulted from hits deposited as part of a Bioproject at NCBI.

```
Hydractinia symbiolongicarpus      ------------------------------------------------------------	0
Hydra vulgaris                     ------------------------------------------------------------	0
Nanomia bijuga                     ------------------------------------------------------------	0
Dynamena pumila                    ------------------------------------------------------------	0
Clytia hemisphaerica               ------------------------------------------------------------	0
Atolla vanhoeffeni                 ------------------------------------------------------------	0
Haliclystus sanjuanensis           ------------------------------------------------------------	0
Craterolophus convolvulus          ------------------------------------------------------------	0
Copula sivickisi                   ------------------------------------------------------------	0
Chironex yamaguchii                ------------------------------------------------------------	0
Chrysaora quinquecirrha            MIWFVLNIISYVLRRFRRETRQFALKSPQDVADAGNRTEFEFRSPAWRPRHSRSPRKSPG	60
Cassiopea xamachana                ------------------------------------------------------------	0
Rhopilema esculentum               ------------------------------------------------------------	0


Hydractinia symbiolongicarpus      -------------------------------MLWYVVSIASYLYQKVMVFVNRQAAIAPR	29
Hydra vulgaris                     -------------------------------MIWYLLSFASHLYRTVSIAFQWRAYDFKE	29
Nanomia bijuga                     ------------------------------------------------------------	0
Dynamena pumila                    -------------------------------MFWFLTSITSYYFQKFLHAIRVLNQIYQK	29
Clytia hemisphaerica               -------------------------------MIWFLTSFTTFYFQKFINVIRVLNRAYVR	29
Atolla vanhoeffeni                 -------------------------------MIWYILSVLSYFIRGLRRRYHP--IQGPA	27
Haliclystus sanjuanensis           ------------------------------------------------------------	0
Craterolophus convolvulus          ------------------------------------------------------------	0
Copula sivickisi                   -------------------------------MIWLVISILSSFIRRLTGREKYITY----	25
Chironex yamaguchii                ------------------------------------------------------------	0
Chrysaora quinquecirrha            VDFRPSQTRRSKCASQIITAFRSRSFQPITEMIWFVLNIISYVLRRFRRETRQFAIKSPQ	120
Cassiopea xamachana                -----------MSAINLSSRGKYNCHAKTRNLPYKITTEELYD--IFGKYGAIRQIRLGR	47
Rhopilema esculentum               -------------------------------MLWFILNMVAYVMRKFRREPRRAALIETD	29


Hydractinia symbiolongicarpus      ----SRRRT--T--------------NSDSAFSDDV---DAPMLRRPR--YGRRLKKKMS	64
Hydra vulgaris                     ----SENED-LSEKYGSFYPS--------------KRTSNASIKFDGS--KKMRLGRTMS	68
Nanomia bijuga                     ------------------------------------------------------------	0
Dynamena pumila                    ----ESLKQ-SKGNY----SVKFK-ANTDF-GEQDEDEEDGRGAWYKK--RHKRFGRTMS	76
Clytia hemisphaerica               ----EKKNE-YENLDSKISTLRFE-NEPSVMFEDDLVCEDQPDGSNQS--RRSRLSRKMS	81
Atolla vanhoeffeni                 AIPNVESEQGHDLYGGSSS--RFIRPF-ARTT-----HDLAPLSYYDP---AKVSNGKES	76
Haliclystus sanjuanensis           ------------------------------------------------------------	0
Craterolophus convolvulus          ------------------------------------------------------------	0
Copula sivickisi                   --SDGGKRKTQLNEKGDPVTPRATRSVTFRRF-----VSDIPESYVEP---VIMAQRKES	75
Chironex yamaguchii                -----------------------------------------------------MALRKES	7
Chrysaora quinquecirrha            DVADAGNR--PEFEFRSPAWRPRQPRSPRKSP-----SAGPPETYFINKRNGRG--RKES	171
Cassiopea xamachana                MFP-------VSPDWRSPSFRT--RPSPKKSF-----RSEPPDTFFTRNELGRGLMRRQS	93
Rhopilema esculentum               GFPVSDGRAGYKGHWRSPGK----RATPRKGF-----SNEPPESFFTRTGPGRKL-RRES	79


Hydractinia symbiolongicarpus      FASGVDGIGQSIAILTSGGDSQGMNSAIRATVRLALHAGAKTFVVYWGYQGLVDGGKNIK	124
Hydra vulgaris                     LSGGVDGTGQCIAVLTSGGDSQGMNAAIRATIRMGIYAGAEMYAVHWGYQGLVDGGDNIK	128
Nanomia bijuga                     ----IEQPNQAVAILTSGGDAQGMNAAIRATARMVLYAQARVYAVYWGYQGLVDGGDSIK	56
Dynamena pumila                    FAGGVDGTGQSIAILTSGGDSQGMNAAIRATTRMALFAGAKVYAVYWGYQGLIDGGDNIK	136
Clytia hemisphaerica               FAGGVDGTGQSIAILTSGGDSQGMNAAIRATTRMALYAGAKVYAVYWGYQGLVDGGENIK	141
Atolla vanhoeffeni                 F-VSVHGSGKSIAILTSGGDSQGMNAAIRATARLGIYAGARVYAVYWGYQGLVDGGEYIK	135
Haliclystus sanjuanensis           ------------------------------------------------------------	0
Craterolophus convolvulus          ------------------------------------------------------------	0
Copula sivickisi                   M-VNVEGTGKSIAVLTSGGDAQGMNAALRAVVRMGIYAGARVYAVYWGYQGLVDGGDNIK	134
Chironex yamaguchii                M-VSVEGTGKRIAVLTSGGDAQGMNAALRAVVRMGIYAGARVYAVYWGYQGLVDGGDNIK	66
Chrysaora quinquecirrha            F-VSVEGSGKSIAVLTSGGDAQGMNAAIRAVARIGIYAGARVYAIYWGYQGLVDGGDNIK	230
Cassiopea xamachana                F-VEVEGSGKSVAVLTSGGDAQGMNAAIRAVARFAIYAGARAYAVYWGYQGLVDGGDSIK	152
Rhopilema esculentum               F-VNVEGTGKSIAVLTSGGDAQGMNAAIRAVARLAIYSGAKAYAVYWGYQGLVDGGDNIK	138


Hydractinia symbiolongicarpus      EIFWDGVHGIIHQGGTVIGSARCTDFRERAGRKKAAYNLIKRGITNLVVIGGDGSLTGAS	184
Hydra vulgaris                     KVCWEDVSGILNKGGTVIGTARCQDFRDRKGRKKAALNLVQRKISNLVVIGGDGSLTGAD	188
Nanomia bijuga                     EFTWDDVNGIIHKGGTVIGSARCQDFRTHEGRRKAVFNLVKRGITNLVVIGGDGSLTGAN	116
Dynamena pumila                    QFSWDDVHGIIHKGGTIIGSARCKDFRDRDGRRKAALNLVKRGITNLVVIGGDGTLTGAN	196
Clytia hemisphaerica               RFHWDDVHGIIQKGGTVIGSARCKDFRNREGRRKAALNLVQRGISNLVVIGGDGSLTGAD	201
Atolla vanhoeffeni                 EMTWDSVSGIIHLGGTVIGSARCKDFRGREGRRKAAYNLVSRKITNLVVIGGDGSLTGAN	195
Haliclystus sanjuanensis           ------------------------------------------------------------	0
Craterolophus convolvulus          ------------------------------------------------------------	0
Copula sivickisi                   EMEWDSVSNIIQLGGTVIGSARCKDFRTKEGRRKAAYNLLVKGITNLVVIGGDGSLTGAD	194
Chironex yamaguchii                EMKWESVSNIIQLGGTVIGSARCKDFRTRDGRRKAAFNLVVKGITNLVVIGGDGSLTGAD	126
Chrysaora quinquecirrha            ELNWESVTGINQLGGTVIGSARCKDFRTREGRKKAAHNLVSRGITNLVVIGGDGSLTGAN	290
Cassiopea xamachana                ELEWQSVTGITHLGGTVIGSARCKDFRTREGRRKAAFNLVSRGITNLVVIGGDGSLTGAN	212
Rhopilema esculentum               ELEWQSVTGITHLGGTVIGSARCIDFRSREGRRQAAFNLVTRGITNLVIIGGDGSLTGAN	198


Hydractinia symbiolongicarpus      LLSSEWKSLVAELKNEGKITEEQAIDHSYLSLVGMVGSIDNDFCGTDMTIGADSALHRVL	244
Hydra vulgaris                     IFRKEWPSLIQELLAEGSITETDSEMCPYLSIVGMVGSIDNDFCGTDMTIGADTALHRIL	248
Nanomia bijuga                     LFRQEWSVLLKELVDLGKVSAEEMIKHSYLNIVGMVGSIDNDFCGTDMTIGADSALHRIL	176
Dynamena pumila                    IFRQEWKSLLDELVVSGETTQQEADQYHFLNIVGMVGSIDNDFCGTDMTIGADSALHRIL	256
Clytia hemisphaerica               IFRHEWKSLLEELVLSGDITQESVENFGYLNIVGMVGSIDNDFCGTDMTIGADSALHRIL	261
Atolla vanhoeffeni                 LFRQEWPELVAKKKEKKE------------------------------------------	213
Haliclystus sanjuanensis           ------------------------------------------------------------	0
Craterolophus convolvulus          ------------------------------------------------------------	0
Copula sivickisi                   LFRKEWPELLNELLEEGKIEKTTAIRHAHLNIVGMVGSIDNDFCGTDMTIGADSALHRII	254
Chironex yamaguchii                LFRQEWPDLLKELTSEGKIDHDTADLYNHLNIVGMVGSIDNDFCGTDMTIGADSALHRII	186
Chrysaora quinquecirrha            TFRKEWPDLLAELAQEGQITRGNAELYGSLNIVGMVGSIDNDFCGTDMTIGADSALHRIV	350
Cassiopea xamachana                EFRKEWPELLKELVDEGKITVGTSQLYSYLNIVGLVGSIDNDFCGTDMTIGADSALHRII	272
Rhopilema esculentum               EFRQEWPELLKELVEEGRIKAGEAVLHGHLNIVGMVGSIDNDFCGTDMTIGADSALHRIV	258


Hydractinia symbiolongicarpus      ESVDAIATTAQSHQRAFILEVMGRRCGYLALIAGISCNADWVFLPEWPSEDGWEENLCDN	304
Hydra vulgaris                     ENVDAISTTAESHQRAFILEVMGRNCGYLALVAGISSGADWIFIPEWPPEDGWESELCEN	308
Nanomia bijuga                     ESADAITTTAASHQRAFVLEVMGRNCGYLALMAGISCSADWVCIPEWPMKDGWQEALCKN	236
Dynamena pumila                    ESVDTIVTTARSHQRAFILEVMGRACGYLALVAGISCTADWIFIPEWPLEDGWEDVLCTN	316
Clytia hemisphaerica               ESVDTIVTTAQSHQRAFILEVMGRDCGYLAMIAGISSGADWIFIPEWPCEEGWEENLCNH	321
Atolla vanhoeffeni                 ------------------------------------------------------------	213
Haliclystus sanjuanensis           ------------------------------------------------------------	0
Craterolophus convolvulus          ------------------------------------------------------------	0
Copula sivickisi                   EAVDAITVTAESHQRAFVLEVMGRHCGYLALAAGIGCGADWIFIPECPPDAGWEQHLCDH	314
Chironex yamaguchii                ESVDAITTTAESHQRAFVLEVMGRHCGYLALAAGIGCGADMIFIPECPPDQGWEADMCDY	246
Chrysaora quinquecirrha            ESVDAISTTAESHQRAFVLEVMGRHCGYLALVAGISVGADWIFLPEWPVEDGWEHHLCQQ	410
Cassiopea xamachana                EAVDAISTTAESHQRAFVLEVMGRHCGYLALVAGIGVGADWIFIPEWPVEDGWESCLCEQ	332
Rhopilema esculentum               ESVDAISTTAESHQRAFVLEVMGRHCGYLALVAGISVGADWIFIPEWPVEDDWQDNLCTQ	318


Hydractinia symbiolongicarpus      LRKTRETGRVSTIIILAEGAQDVHGNPVTAAQVKELLS-----KKLKYDTRVTVLGHVQR	359
Hydra vulgaris                     LKKMRSRGRLLTIIIISEGAQDIHGNPITANKVKELLT-----NSLKLDTRVTILGHVQR	363
Nanomia bijuga                     LKRGRESGKLTTIVIVAEGAQDVHGKSVTSNEIKEMLS-----QQLKLDTRVTVLGHVQR	291
Dynamena pumila                    LQRNRENGKQLAILIIAEGAHDAKGFPITADDVRKLLA-----TRLDLDVRATVLGHVQR	371
Clytia hemisphaerica               LQKSRKSGRLLAVLIIAEGAHDSKGNHISANDVKNLLK-----TRLELDVRTTVLGHVQR	376
Atolla vanhoeffeni                 ------------------------------------------------------------	213
Haliclystus sanjuanensis           -----------------------------------------------------------R	1
Craterolophus convolvulus          ------------IIILAEGARDCHGNSITSQKVKEAMTCRRSPNEMLIDTRITVLGHVQR	48
Copula sivickisi                   LKLSRDKGRLSSIIIVSEGATDTEGKSITANQVKCLLS-----ERLGFDTRVTVLGHVQR	369
Chironex yamaguchii                LKKSRDTGRLSSIIIVSEGATDKNGKCISAGYIKEILS-----NRLGYDTRVTVLGHVQR	301
Chrysaora quinquecirrha            LRKGRETGRLLSIIILAEGAHNVHGEPVTAGHVKNLIA-----DKLGYDTRVTVLGHVQR	465
Cassiopea xamachana                LKKSRDGGRLLSIIILAEGAQNVNGEPVTANQVKNLIS-----KNLGYDTRVTVLGHVQR	387
Rhopilema esculentum               LRKGRENGRLLSIIILAEGAQNIHGEPVTANQVKDLIC-----KNLGYDTRVTVLGHVQR	373


Hydractinia symbiolongicarpus      GGTPSAFDRLLASRLGAEAALCVLESG-EQTIPTVMCLNGQQIVKKPLQSCVDSCIEIKT	418
Hydra vulgaris                     GGRPSAFDRILASRLGCEAALSIIEAN-VASEPMVMCLEGSQIIARPLHQCVEWCNEIKA	422
Nanomia bijuga                     GGTPSAFDRLLASRFGAEAALCVLEAN-ESSEAFVMCLCGSQIVKKPLSECVEQCNGIKQ	350
Dynamena pumila                    GGTTSAFDRLLASRLGAEAALAVLEKG-EGSEASVMCLCGQQIIQKPLNQCVSECNNIKK	430
Clytia hemisphaerica               GGTTSAFDRLLASRLGAEAALTVLEAD-EDSEASVMCLCGQQIVKKPLHECVDMCNQIKA	435
Atolla vanhoeffeni                 ------------------------------------------------------------	213
Haliclystus sanjuanensis           GGSPSAFDRVLATRFGAEAALTVLEAG-PDTPSYVMCLEGQLIVKRSLSQCVDKCNRIKA	60
Craterolophus convolvulus          GGSPSAFDRILATRFGAEAALTVLESN-ADTPSYVMCLQGQQIVKKSLSECVEQCNKIKE	107
Copula sivickisi                   GGAPSAFDRLLSCRLGAEAALTVLEGT-ETSPSYVICLEGNRMARKSLNECVEMCNKIKK	428
Chironex yamaguchii                GGSPSAFDRLLSCRLGAEAALTVLEGT-QTSPSYVICLEGNKMVRKPLNVCVESCNKIKA	360
Chrysaora quinquecirrha            GGAPSAFDRLLATRLGAKAALTVLEDTDNDKPPQVVCLDGNQIVKKSLMECVEQCDAIKV	525
Cassiopea xamachana                GGAPSAFDRVLSMRLGAEAALTVLEDD-GEKPPQVMCLEGNLIVKKSLLENVEKCNAIKK	446
Rhopilema esculentum               GGAPSAFDRLLAMRLGAEAALTVLEDV-KDMPPQVVCLDGNQIVKKNLMENVEKCNAIKK	432


Hydractinia symbiolongicarpus      AFDNGEFEKVVKLRGSTFEKNLIMFKELKHVSPPQTPTNKGL------------------	460
Hydra vulgaris                     AYNEKNFKKVVELRGSSFQTSLEMYTILKHASFPTE----SK-----QGYTLGVINVGAP	473
Nanomia bijuga                     AYAERNFERVIELRGKSFRNNLEIFKKFKKHSPRND---KET-----SGFRFGLINCGAP	402
Dynamena pumila                    AYLEKDYKGVIQLRGRSFQTSLEMYRKLKHAIPPKSITDSPS-----QKFTFGVINIGAP	485
Clytia hemisphaerica               AYKDRNFDKVIELRGKSFVSSLEMYKKMKHVKAPDSKHK--M-----GGYTFVIMNIGAP	488
Atolla vanhoeffeni                 ------------------------------------------------------------	213
Haliclystus sanjuanensis           AFEAKDYDEVVSLRGRSFQRNLSLYKTLRKFQPPMLDKCKDDNSLCKKTYTLAVLNVGAP	120
Craterolophus convolvulus          AFDAKDFDEVVSLRGRSFQRNLSLYKTLRKLEPPSLEECKEA-GNCRKSFTMAVLNVGAP	166
Copula sivickisi                   AYDDKDFKVIEELRGRSFKRNLEAFHTLKRLKPPKSSHCTID-GVCE-GYSLAVMNVGAP	486
Chironex yamaguchii                GYEAKEFTAVEQLRGRSFKRNLEAFYTLKLLKPPKESHCTTD-GRCE-GFSLAVMNVGAP	418
Chrysaora quinquecirrha            AYEQRDFKKIVELRGGSFRRNLEAYKTLKLLCPPEGSHCHSD-GTCDEGYMMAIMHVGAP	584
Cassiopea xamachana                AYAEKDFKKVVELRGASFSRNLQAYKVLRQLEPPTDAHCHPD-GSCDQGYTLAVMHVGAP	505
Rhopilema esculentum               AYAEKDFKKVVELRGASFKRNLAAYKVLKLFDP---TQCNTD-GTCKMGYTLAVMNVGAP	488


Hydractinia symbiolongicarpus      ------------------------------------------------------------	460
Hydra vulgaris                     AGGMNAAVRSFVRTALYHGHTVLGITNGFDGLLENSVHTMTWSEVDKWTEIGGCMLGVTR	533
Nanomia bijuga                     AGGMNAANASFVKAALYNGHTVIGFRDGFTGVLENKFVDMSWHEVGEWPALGGSKLGVSR	462
Dynamena pumila                    AGGMNAAIGAFVRSALYEGHRVIGIEDGFEGLLKNQVHEMTWHEVEEWAGMGGSNLGVTR	545
Clytia hemisphaerica               AGGMNAAVSAFVRSALYDGHKVLGVKDGFEGLLTDEVHYMTWSEVDMWAGLGGSRLGVTR	548
Atolla vanhoeffeni                 ------------------------------------------------------------	213
Haliclystus sanjuanensis           AGGMNQAVRSFVRSALFAGHRVIAFRDGFKGLFQGLVQKMEWNDVDRWGSDGGSNLGTYR	180
Craterolophus convolvulus          AGGMNQAVRSFVRSSLFAGHRVIGFRDGFQGLLKDMAEEMKWNDVDKWGSAGGSNLGTYR	226
Copula sivickisi                   AGGMNPGVRAFVRSALCNGHSVWGIKDGFDGLIKGQIDIMGWADVDKWSSAGGSNLGTTR	546
Chironex yamaguchii                AGGMNPAVRGFVRSALCDGHSVWGIKDGFEGLVRGDIDLMGWSDVDKWSSHGGSNLGTTR	478
Chrysaora quinquecirrha            AGGMNPATKAFVRSSLNAGHSVLAIKDGFDGLVRGAVNLMGWNDVDRWSGLGGSNLGVTR	644
Cassiopea xamachana                AGGMNPATKAFVRDALTKGHSVVAINDGFDGLVKGQIRLMGWNDVDKWSNLGGSNLGVTR	565
Rhopilema esculentum               AGGMNPATKACVRSALNQGHSVLAISDGFDGLIKGQVRLLGWNDVDKWSNLGGSNLGVTR	548


Hydractinia symbiolongicarpus      ------------------------------------------------------------	460
Hydra vulgaris                     TLPDN-DLEKIHRRLKEHHIDGLFIVGGFEAFVSLIQLTEARSSYSAFRIPMVLVPATMS	592
Nanomia bijuga                     TLPNE-NIEKIRDRFCEHKLSALFIIGGFEAFNSLLQLYEARSKYEEFQIPMVLVPATMS	521
Dynamena pumila                    LLPGN-HFEEIGERFKEHNLNALLLVGGFEAFHGLMQLTEARCDYEEFCIPMVLIPATMS	604
Clytia hemisphaerica               TLPGK-HLNEVSQVFKNHNVKGLLIIGGFEAFHSLVQLTDAREQYKEFCIPMVLVPATMS	607
Atolla vanhoeffeni                 ------------------------------------------------------------	213
Haliclystus sanjuanensis           NLPSE-NLDLVAEQFRVNSINGVLIIGGFESFTSALQLTEARRSFKEFRIPIVVLPATMS	239
Craterolophus convolvulus          TLPSE-NYDLVAERLTNYNIKGLLIVGGFEGYSAALQLAEARQSYEAFRIPIVVIPTTMS	285
Copula sivickisi                   TLPST-NFAAVAKNLEKFRIKGILIIGGFEAYHATLELANARKNYRAFNIPIVNLPATLS	605
Chironex yamaguchii                TLPSE-NFDAIARNLENFRIKGLLVIGGFEGFRSVLELAEARTKYKAFNIPIVLIPATLS	537
Chrysaora quinquecirrha            TLPEKAGFDAIAAQLSSKGIQGLVIIGGFEAFTSALQLAKARSSYKPFRIPIIVLPATMS	704
Cassiopea xamachana                TLPESVGLENINSRLGEHGIQGLLIVGGFEAFHSALQLAKAKDLFKSLRIPIIVIPATMS	625
Rhopilema esculentum               TLPSKAGLAAVNDRLSEFGIQGLLIVGGFEAFNSALELAKGRERQKAFRIPIIVIPATMS	608


Hydractinia symbiolongicarpus      ------------------------------------------------------------	460
Hydra vulgaris                     NNVPGTWFSLGADTTLNVIVECCDKIRQSAGSTARSVFIVETMGGKCGYLPTMAAIAAGA	652
Nanomia bijuga                     NNVPGCWLSLGADTTLNVIVESCDRLRQSASSSFNRVFLVETMGGKCGYLATMAGIAGGA	581
Dynamena pumila                    NNVPGTWFALGADTTLNVIVECCDRLRQSAHSSKDRVFIVETMGGKCGYLATMAAIAGGA	664
Clytia hemisphaerica               NNVPGTWFSLGADTTLNVIVESCDRIRQSASSSRDRVFIVETMGGKCGYLATMAAIAGGA	667
Atolla vanhoeffeni                 ------------------------------------------------------------	213
Haliclystus sanjuanensis           NNVPGTYFSLGADTTLNVIIEACDRVKQSATSSQGRVFVVETMGGKCGYLASMAGIAVGA	299
Craterolophus convolvulus          NNVPGTYFSLGADTTLNVIVEACDRVRQSATSSQGRVFVVETMGGKSGYLATMAGIAVGA	345
Copula sivickisi                   NNVPGTHFSLGADTTLNVIVESIDRIKQSASSSQDRVFLIETMGGKCGYLATMAAIAGAA	665
Chironex yamaguchii                NNVPGTHFSLGADTTLNVIVECCDRIKQSASSSQDRVFLVETMGGKCGYLATMAGIAGAA	597
Chrysaora quinquecirrha            NNVPGSHFSLGADTTLNVIVEACDRIRQSASSSQDRVFVVETMGGKCGYLATMGGIAASA	764
Cassiopea xamachana                NNVPGTHFSLGADTTLNVIVESCDRIRQSASSSQDRVFVVETMGGKCGYLATMAGIAAAA	685
Rhopilema esculentum               NNVPGTHFSLGADTTLNVIVESCDRIRQSASSSQDRVFVVETMGGRCGYLATMAGIAAAA	668


Hydractinia symbiolongicarpus      ------------------------------------------------------------	460
Hydra vulgaris                     DSAYIFEECLTLEELKRNIDHLKNKFTNSGLR-RGLVLRSEHCNSNYTTDFITRLYSEEG	711
Nanomia bijuga                     DSAYIFEEKFTLADLKKNIEHLKNKFTNKK-SHRGLILRTEQCNDNYSTEFITRLYQEEG	640
Dynamena pumila                    DSAYIFEDHFTLDDLKSNIKHLIERFKYSKFG-RGLILRAENCNENYDTDFINRLYSEEG	723
Clytia hemisphaerica               NAAYIFEDHFTMVDLKKNIQQLVHRIKNSEFQHSGLILRAENCNENYTTDFITRLYAEEG	727
Atolla vanhoeffeni                 ------------------------------------------------------------	213
Haliclystus sanjuanensis           DSAYVYEEPFNVDTLQNDVQHLLSKFHHYNIN-RGLVLRNEKCNERYTADFITSLYEEEA	358
Craterolophus convolvulus          DSAYIFEESFNAKSLQNDIQHLLSKFKRYNIK-RGLVLRNEKCNARYTTDFITSLYEEEA	404
Copula sivickisi                   DSAYIFEEEFGAPDLLADIQHLVYKFQNSGIK-RGVVLRAEGCSKRYTADFITAMYEEEA	724
Chironex yamaguchii                DAAYIFEEKFSAADLLADIQHIIYKFKHSGIK-RGVVLRAEGCSKLYTADFITAMYEEEA	656
Chrysaora quinquecirrha            DAAYIREEPFGIDELQADVQHLIYKFQHTSIK-RGLVLRAQEANERYTCKFITALYEEEA	823
Cassiopea xamachana                DAAYIREEPFGVYELQADCNHLIYKFQNTGIK-RGLVLRSQEANDRYTTKFITALFEEEA	744
Rhopilema esculentum               DAAYIREEPFGVYELLADANHLMYKFQNTGIK-RGLVLRAQEANERYTTKFITSLYEEEA	727


Hydractinia symbiolongicarpus      ------------------------------------------------------------	460
Hydra vulgaris                     KKLFVVRSNVLGHIQQGGSPSTFDRLYGTRYGVKALHFLLQKIEEA-KDEYGLVNATSSD	770
Nanomia bijuga                     QGLFDARPCVLGHTQQGGAPSPFDRLTGTRSGIRAFEFLHKKMTSSQKKAAGIVRTNDAE	700
Dynamena pumila                    KGVFVTRTNILGHVQQGGTPTPFDRLCGTRHAVRAYSYLLEKIQEN-VNENGVVRTYTDD	782
Clytia hemisphaerica               KGVFTTRQNVLGHVQQGGVPTPFDRLCGTRQAFKSLNFLVEQIEKH-LDEEGNVCTTTKE	786
Atolla vanhoeffeni                 ------------------------------------------------------------	213
Haliclystus sanjuanensis           KGIFITRKIVLGHMQQGGKPSPFDRMVATRQARRAMEFLVEHIDAN-IDENGCIEATTEE	417
Craterolophus convolvulus          QGSFITRKLVLGHIQQGGPPSPFDRMVATRQARRAMEFLVKCIEEN-LDEFGCVQATAED	463
Copula sivickisi                   AGTFITRKAVLGHVQQGGPPSPFDRVNATRQAMKAMKFLEVQIREN-TDENGTLHCSQPE	783
Chironex yamaguchii                KGVFITRKNVLGHVQQGGPPSPFDRLNGTRQAMKAMKHLVTQIKQC-SDASGVVFASQPE	715
Chrysaora quinquecirrha            KGVFITRENVLGHLQQGGPPSPFDRLCGTRNAMKAVHFLISKIKEN-LDENGQVSATTSD	882
Cassiopea xamachana                KGVFITRESVLGHLQQGGPPSPFDRLNGTRQAVKAVRFLIEKIGEN-ISDNGIVTATAPD	803
Rhopilema esculentum               KGVFITRESVLGHLQQGGPPSPFDRLNGTRQAMKAVRFLIENIGEN-IDENGIVSATSPN	786


Hydractinia symbiolongicarpus      ------------------------------------------------------------	460
Hydra vulgaris                     SSCVLGHIKNHSQFTPVLDLKDKADFKNRLPKHYWWKRTRPLLKVLAQYKVMFAREVESH	830
Nanomia bijuga                     SACVLGIIRNQSVFTPVVELKDVADFKNRLPKECWWMNMRPLLKVLARYHTVYHREEEL-	759
Dynamena pumila                    SSCVLGHIKNHSRFTPMAELKEQTDFKNRLPKEYWWKSIQPLLRILANYQVMYNRESQVY	842
Clytia hemisphaerica               SSCILGHIKNHSQFVPISDLKEKTDFVNRLPKEQWWRSIQKLLKIFARYEIAYDREDTAT	846
Atolla vanhoeffeni                 ------------------------------------------------------------	213
Haliclystus sanjuanensis           TACVLGIVKSSTTFTPLQELKKETDFKNRIPKANWWRDIRKLLRVMAKHNEAFQRESSIS	477
Craterolophus convolvulus          TACILGIIKTATTFTPVQSLKMETDFKNRIPKYNWWLDVRPLLRIMAQHKEVFHREASVL	523
Copula sivickisi                   TACVLGIVYSQTMFRPVQELAEETDFKNRLWNERWWFGIRPLLRILARHKEVYTREAVAE	843
Chironex yamaguchii                TATVLGILYSQTAFTPVQELKDEVDMTNRLWNESWWSGIRPLLRILARHKEIYTREVESD	775
Chrysaora quinquecirrha            SACVLCHVKAQTLFRPVEELEFEADMKHRVAKDNWWLKLRPLLRVLARHKEVFPGEDDS-	941
Cassiopea xamachana                TASVLCHVKAQTLFRPVEELEKEADMKNRVPKDNWWLVVRPLLRVLARHKEIFTREA---	860
Rhopilema esculentum               TACVLCHVRAQTLFRPVVELEAEADMKNRVPLNNWWLVVRPLLRVLARHRETFIREVDE-	845


Hydractinia symbiolongicarpus      -------------------------	460
Hydra vulgaris                     ADEISIKNYIDEVEVEAFVDDMKMP	855
Nanomia bijuga                     -------------------------	759
Dynamena pumila                    E------------------------	843
Clytia hemisphaerica               -------------------------	846
Atolla vanhoeffeni                 -------------------------	213
Haliclystus sanjuanensis           L------------------------	478
Craterolophus convolvulus          P------------------------	524
Copula sivickisi                   S------------------------	844
Chironex yamaguchii                D------------------------	776
Chrysaora quinquecirrha            -------------------------	941
Cassiopea xamachana                -------------------------	860
Rhopilema esculentum               -------------------------	845
```

**SM3**. **Multiple alignment of Medusozoan phosphofructokinase (PFK) protein sequences**. The two conserved PFK signatures are shaded in gray; the residues involved in both the active site residues and in the ADP/phosphate binding are shaded in black. In yellow the conserved pfkB domain (Poorman et al., 1984; Chen et al., 2017; Shullia et al., 2019). Accession numbers are not provided since sequences resulted from hits deposited as part of a Bioproject at NCBI.

```
Hydra vulgaris                     ---------------MSGSKARLEYKEEVPV--------TYPFAMTRGEYLSLLNIDQPP	37
Dynamena pumila                    ---------------MSGSVARSEYLNEYSSRR----QKQQGNAVNFREFLCNINVDTIP	41
Clytia hemisphaerica               ---------------MSGQENRTEYLKDAFKRR----TLS--TAKTVLEYHSKINIDETA	39
Copula sivickisi                   MEEIVFEEDRERSRTMSGSKARFEYLEDLQKRR---DQMETAPARNMLEHRCAIDVDLTP	57
Chironex yamaguchii                ---------------MSGSKARFEYLEDIEHRR---NQLQMPSAHTMLEHRCGLDVDLTP	42
Haliclystus sanjuanensis           ---------------MSGKVARQEYQHDIVQRD---AQSQESKARNFMEHTCALNVDAVP	42
Craterolophus convolvulus          ---------------MSGRTTRDEYQKDISQRQ---TLDQFAQARNYKEHTCSLNVDAVP	42
Hydractinia symbiolongicarpus      ---------------MSGSSARHEYRRDLTRHD---SLVTFPDASTWREYLCYLDIDDSP	42
Atolla vanhoeffeni                 ---------------MSGKSARFEYLKDIDRRKDHHAQMQAAESNTMMEFRSALDVDSVP	45
Nanomia bijuga                     ------------------------------------------------------------	0
Chrysaora quinquecirrha            ---------------MSGKVARFEYKEDMAERD---AKLNVAHARNHLEFTCALDVDSVP	42
Cassiopea xamachana                ------------------------------------------------------------	0
Rhopilema esculentum               ---------------MSGKVARFEYNEDMVARD---ALLNATHARNHLEFCCGLNIDTVP	42


Hydra vulgaris                     TVIRGTGIICTIGPSSQEVSVLEELIANGLNIARINFSHGSYEYHQKTIDNIRIAAKKIF	97
Dynamena pumila                    PAVRAVGIICTIGPACQTVEKLEQLNASGMNIARLNFSHGTYEYHQQTIDNVRVASAKTA	101
Clytia hemisphaerica               PVIRSVGIICTIGPTSQKVEQLEALISAGMNIARMNFSHGTHEYHGSTIENVRAAAAKVY	99
Copula sivickisi                   LVVRGTGIICTIGPSCNSVEKLKELIMNGMNIARMNFSHGTHEYHKGVIDNVREAAKVEH	117
Chironex yamaguchii                LVVRGTGIICTIGPSCKDIEKLKMLIMNGMNIARMNFSHGSHEYHKEVLDNVRKAAEVEH	102
Haliclystus sanjuanensis           NVVRGTGIICTIGPVCNTVEKLKDMICNGMNIARLNFSHGSHEYHQSVIDNVREAAKEEE	102
Craterolophus convolvulus          FVVRGTGIICTIGPVCNTVDKLKEMVSIGMNIARLNFSHGSYEYHQGVIDNVREATKEDP	102
Hydractinia symbiolongicarpus      PVVRGTGIICTIGPACQSVENIKDLIASGMNIARMNFSHGTYEYHQDTIDNVRAAAAASF	102
Atolla vanhoeffeni                 PVVRGTGIICTIGPACQSVKQLQELIHSGMNIARMNFSHGTHEYHKQTIDNVREAAKVEY	105
Nanomia bijuga                     ------------------------------------------------------------	0
Chrysaora quinquecirrha            PVVRGTGIVCTIGPACQSVEKLKEMIYSGMNIARMNFSHGTHEYHKKTIDNVREAAKVEY	102
Cassiopea xamachana                ------------------------------------------------------------	0
Rhopilema esculentum               PVVRGTGIICTIGPACQDVDKLKEMIYSGMNIARMNFSHGTHEYHKKTIDNVREAAKVEH	102


Hydra vulgaris                     PHVVGLALDTKGAEIRTGNIKNGGE--VTYVKGQELKVSSDPYFKNQCSESLLFLDYPDL	155
Dynamena pumila                    PHPVAIALDTKGPEIRTGMMKSGGD--ITYQTGQDLRVSCDPALREQCDENQQFLEYSSL	159
Clytia hemisphaerica               PHPIAIALDTKGPEIRTGMMKSGKD--VTYKKGQALKISCNPAMREQCDENQQYLEYPSL	157
Copula sivickisi                   PHPLAIALDTKGPEIRTGLTKGNVD--VMYKRNSIIKVTNDKAFAESGDENTIYVDYAGL	175
Chironex yamaguchii                PHPVAIALDTKGPEIRTGLIKGNGE--VTFKPGSELKLTNDKKYYEIGDEKTLYLDYAGL	160
Haliclystus sanjuanensis           PHPVAIALDTKGPEIRTGLVKNDED--VMFVRGSDLKLTTDQAFREIGDAKTLYLDYADL	160
Craterolophus convolvulus          SHPVAIALDTKGPEIRTGLVKDNVD--VDFVRGSTLKLTTDKAYREIGDASTLYLDYEDL	160
Hydractinia symbiolongicarpus      PHPVGLALDTKGPEIRTGNMKDGGT--ASYKKGQELKVTCDPALKEECGVDLQYLEYPSL	160
Atolla vanhoeffeni                 PHPVALALDTKGPEIRTGVMKSGGD--TTYNKGQMLRVTTDDALKEQCDENLLYIDYHGL	163
Nanomia bijuga                     ------------------------------------------------------------	0
Chrysaora quinquecirrha            PHPVAIALDTKGPEIRTGIMKAGLDTETKYEKDQVLKLTIDENFREQCDKELLYIDYVGL	162
Cassiopea xamachana                ------------------------DTETQYKKGQSLKITTDDKLREQCDENLLYVDYHSL	36
Rhopilema esculentum               PHPVAIALDTKGPEIRTGIMKAGTDSETTYKKGAALRVTTDLSLREQCDENLLYVDYKGL	162


Hydra vulgaris                     IHSVKRGSKIVIADGNFLLEVKDIIDNNNLLAVVLNDATIGNRKNCNLPGAVVVLPAVSE	215
Dynamena pumila                    CKSLKPGNKIFIADGMLALEVKKIVDDKTLVATVLNEATIGSRKNCNLPGAIIDLPAVSE	219
Clytia hemisphaerica               CKSLKAGSKIFIADGMLILEVREVLNDDMLMAEVKCDATIGSRKNCNLPGAIIDLPAVSE	217
Copula sivickisi                   QKSVKPGGKIYIADGMLSLEVQEIVA-DGVKAKVLNDTAIGSRKNCNLPGAIVDLPAVSE	234
Chironex yamaguchii                QTSVKVGGKVYIADGMLSLEVKEVVT-DGVITRVINEATIGSRKNCNLPGAIVDLPAVSE	219
Haliclystus sanjuanensis           RTSVKVGGKIYIADGSLSLKVKEIVA-DGVITEVLNDAALGSRKNCNLPGAVVTLPAVSK	219
Craterolophus convolvulus          TKSVTVGGKIYIADGSLSLKVKEIVK-DGVLAEILNDASLGSRKNCNLPGAVVTLPAVSE	219
Hydractinia symbiolongicarpus      TKSLHKGSKIFIADGLLALEMVKLIDSKTIMTRVLNDATIGSRKNCNLPGAIIDLPAVSE	220
Atolla vanhoeffeni                 MGSVKKGSKIFIGDGILSLEVQEVHD-AHVMALVCNDATIGSRKNCNLPGAIVTLPAVSE	222
Nanomia bijuga                     ------------------------------------------------------------	0
Chrysaora quinquecirrha            PKSVQKGTKILVADGMLSLLVQEIHS-DHVMVKVLNTASIGSRKNCNLPGCIVDLPAVSE	221
Cassiopea xamachana                RKSVRVGSKIFIADGLLSLEVVELND-DHVMAKVLNDAAIGSRKNCNLPGAIIDLPAVSE	95
Rhopilema esculentum               AKSVKKGSKIFIADGLLSLEVTDVLD-DHVMTKILNDAAISSRKNCNLPGAIIDLPAVSE	221


Hydra vulgaris                     KDKQDLLFGVKNKVDMVFASFIRKASDVADVRNALGEEGKSIKVIAKIENYEGVVNIDEI	275
Dynamena pumila                    KDKKDLLFGVQNKVDMVFASFIRKKEDVLEVRAALGEEGKHIKIIAKIENHEGIMNIDEI	279
Clytia hemisphaerica               KDRSDLLFGVEQKVDMVFASFIRKKEDIFDIRKVLGEEGKSIKIIAKVENHEGITNIDEI	277
Copula sivickisi                   KDVSDLKFGVKEGVDMVFASFIRKASDVRDVRKVLGEEGKRIMIIAKIENHEGIKNIDDI	294
Chironex yamaguchii                KDIEDLKFGVKEGVDMVFASFIRKAADVRDVRKVLGEEGKRIMIIAKIENHEGIKNIDEI	279
Haliclystus sanjuanensis           KDIEDLKFGVKNNVDMVFASFIRKASDVNAVREALGVEGKHISIIAKIENHEGIINIDEI	279
Craterolophus convolvulus          KDKEDLKFGVENEVDMVFASFIRKASDIVAVREALGPKGKNILIIAKIENHEGIINIDSI	279
Hydractinia symbiolongicarpus      KDKKDLQFAVQNQVDMVFASFIRKHRDVLEVREALGPNGKDIKIIAKIENHEGIMNIDRI	280
Atolla vanhoeffeni                 KDISDLRFAVEQKVDMVFASFIRNAEGVREVRKVLGEDGKYIRIISKIE-----------	271
Nanomia bijuga                     ------------QLDMIFASFIRKRQDVLDVRAALGETGKDIKIISKIENHEGIVNIDEI	48
Chrysaora quinquecirrha            KDKADLKFGVEQKVDMIFASFIRKAKDILEVKKVLGEEGKNIKIIAKIENHEGILNIDEI	281
Cassiopea xamachana                KDVQDLKFGVKNQVDMIFASFIRKAQDIRDVRKVLGEEGKDIKIIAKIENHEGILNIDEI	155
Rhopilema esculentum               KDVQDLQFGVENEVDMIFASFIRKAQDIRDVRKVLGEKGKDIKIIAKIENHEGIINIDEI	281
                                                :**:******:  .:  :: .**  ** * :*:*:*


Hydra vulgaris                     IAAADGIMVARGDMGMEMPAEKVFLAQKILITRGNRAGKPVICATQMLESMIKNPRPTRA	335
Dynamena pumila                    IATADGVMVARGDMGMEIPLEKVFLAQKLLIAKCNKAGKPVICATQMLESMIKNPRPTRA	339
Clytia hemisphaerica               VEHSDGVMVARGDMGMEIPLEKVFLAQKLIISKCNKNKKPVICATQMLESMIKNPRPTRA	337
Copula sivickisi                   LTEADGIMVARGDMGMEIPLEKVFLAQKMMIARCNLANKPVICATQMLESMVGNPRPTRA	354
Chironex yamaguchii                LTEADGIMVARGDMGMEIPLEKVFLAQKMMIARCNLANKPVICATQMLESMVNNPRPTRA	339
Haliclystus sanjuanensis           LRVADGIMVARGDMGMEIPLEKVFIAQKMLISRCNFACKPVICATQMLESMINSPRPTRA	339
Craterolophus convolvulus          LKEADGIMVARGDMGMEIPLEKVFIAQKMIISRCNLACKPVICATQMLESMVNSPRPTRA	339
Hydractinia symbiolongicarpus      LCYADGIMVARGDMGMEIALEKVFLAQKMIIAKCNRAGKPVICATQMLESMIQNPRPTRA	340
Atolla vanhoeffeni                 ------------------------------------------------------------	271
Nanomia bijuga                     LAVTDGVMVARGDMGMEIPLEKVFVAQKMIIARCRALNKPVICATQVKGVLGVLVRCNS-	107
Chrysaora quinquecirrha            LVEADGVMVARGDMGMEIPLEKVFLAQKMIIARCNMAAKPVICATQMLESMTDNPRPTRA	341
Cassiopea xamachana                LMETDGVMVARGDMGMEIPLEKVFLAQKMIIARCNMAAKPVICATQMLESMTENPRPTRA	215
Rhopilema esculentum               LMETDGVMVARGDMGMEIPLEKVFLAQKMIIARCNMAAKPVICATQMLESMTENPRPTRA	341


Hydra vulgaris                     EITDVGNAVVDGADCVMLSGETTNGAYPIEAVAVMHKICRQAATVQFYKESFSEHLNKN-	394
Dynamena pumila                    EITDVGNAVTDGADVVMLSGETASGTYPVEAVSFMHKICREAASAIHHRRVFEELKLGNE	399
Clytia hemisphaerica               EITDVGNAVTDGCDCVMLSGESANGAYPVEAVTYMHKICKEACSAIHYEKRFQELTASSH	397
Copula sivickisi                   EVSDVANAVLDGADCVMLSGETAKGAYPAEAVLTMHKICREAEAAAFHQMTYEQLESSK-	413
Chironex yamaguchii                EVSDVANAVLDGADCVMLSGETAKGAYPAETVLTMHKICREAEAATFHQMMYDQLKNTK-	398
Haliclystus sanjuanensis           EVSDVANAVLDGADCVMLSGETAKGAYPCEAVAMMHRIAREAERTVFHRQMFENLKSAR-	398
Craterolophus convolvulus          EVSDVANAVLDGADCVMLSGETAKGAYPCEAVAMMHRIAREAETTVFHRHLFNNLKSAR-	398
Hydractinia symbiolongicarpus      EITDVGNAVVDGADCVMLSGETASGAYPIESCAMMHKICREAASAVYHRILYDELKNTK-	399
Atolla vanhoeffeni                 ------------------------------------------------------------	271
Nanomia bijuga                     ------------------------------------------------------------	107
Chrysaora quinquecirrha            EASDVANAVLDGADCVMLSGETAKGAYPSQAVLTMHKICREAEAAIFHRNLYDDLKNAK-	400
Cassiopea xamachana                EASDVANAVLDGADCVMLSGETAKGAYPSQAVLTMHKICREAEAAIFHRALYDDLKNAK-	274
Rhopilema esculentum               EASDVANAVLDGVDCVMLSGETAKGAYPSQAVLTMHKICREAEAAIFHRALYDNLKNAK-	400


Hydra vulgaris                     HVTDATETTCIAAVAASFKALASAIIVLTTSGRTSWIMSKYRPQCPIIVITRNEQVARLC	454
Dynamena pumila                    STADIAETIAISAVSASFQADAAAIIVLTTSGRSAWYLSHYHPSCPIIVITRNSHVARCA	459
Clytia hemisphaerica               S-TDTAEIVALSAVSAAFKVSAAAIIVLTTSGRSATLLSHFCPNCPIIVITRSAPVARKL	456
Copula sivickisi                   SFTDACETTCIAAVSASFKVHAAAVICMSTSGRTAHILSHYRPRCPIIVLTRDAQVARQC	473
Chironex yamaguchii                QFTDACETTCIAAVSASFKVNAAAIICMSTSGRTAHILSHYRPRCPIIVLTRDPMVSRQS	458
Haliclystus sanjuanensis           THTDSTETTCIAAVSASFNLHAAAIIVLTTSGRTAYLLSHYRPMCPIMVLTRNATVARQA	458
Craterolophus convolvulus          LVTDSSETTCIAAVTASFNLNAAAIIVLSTSGRTAHLLSHYRPMCPIIVLTRSGIVARQA	458
Hydractinia symbiolongicarpus      SFTETAETTSIAAVSASFKVNAAAIIVLTTSGKTAAMLAHYHPRCPVLMITRSGHVARCG	459
Atolla vanhoeffeni                 ------------------------------------------------------------	271
Nanomia bijuga                     ------------------------------------------------------------	107
Chrysaora quinquecirrha            SLTDATETTCIAAVSASFKVNAATIIVFSTSGRTARILSHYRPRCPTILITRNEGVARQA	460
Cassiopea xamachana                TLTDATETTCIAAVSASFKVNAAAIISFSTSGTTARILSHYRPRCPIILITRTPRVARQA	334
Rhopilema esculentum               TLTDATETTCIASVSASFKVNAATIIAFSTSGRTARILSHYRPRCPIILITRNKRVCRQA	460


Hydra vulgaris                     HLYRGLFPLVYSTPRIKTWHEDMFDRLEFAITYGKDMGFIKTGSSVVFVSGCQPGTNSTN	514
Dynamena pumila                    HLYRGLFPLEYPAERKGTWTEDLDARLQFGIQKGRDMGFIYEMSKVIFVCGYQPGSSTTN	519
Clytia hemisphaerica               HVYRGLFPLEYPYARKGTWTEDIDARLDFGIKKGLDMGFILPSSKVIFVCGYQPGSSTTN	516
Copula sivickisi                   HLYRGLFPLFYDKDRAPTWSEDIDARLHFAVQTGCNMGFISPGSFIVFVSGYMPGAQTTN	533
Chironex yamaguchii                HLYRGLFPLHYNKPRAATWSEDIDARLIFAVETGTSMGFIAQGSFIVFVSGYIPGTQTTN	518
Haliclystus sanjuanensis           HLYRGIFPLFYNKERASEWSEDIDNRLEFAIQRGREMGFIQLGSSVVFVSGWKPGAKTTN	518
Craterolophus convolvulus          HLYSGLFPIFYNKERAPLWSEDVDDRLEFGIQKGREMGFIKAGSSIVFVSGWRPGAKTTN	518
Hydractinia symbiolongicarpus      HLYQGIFPLEYPFARKNTWSEDMDARLEFGIEKGRDMGFIKKGSFIIFVSGWQPGVGTTN	519
Atolla vanhoeffeni                 ------------------------------------------------------------	271
Nanomia bijuga                     ------------------------------------------------------------	107
Chrysaora quinquecirrha            HLSRGLFPLYYDESREKNWSDDVNARLMFAVKAGRDMGFIRKGSFIVFVVGWSPGSQTTN	520
Cassiopea xamachana                HLARGLFPLYYDEPREKNWSDDVNARLLYAVEKGKDMGFIKAGSFVVFVVGWSPGSQTTN	394
Rhopilema esculentum               HLSRGLFPLYYDEPRQNNWSDDVNARLVYAVTKGKDMGFIRNGSFIVFVVGWSPGSHTTN	520


Hydra vulgaris                     TVQIIQVDEKPISGIRYNTNFCLI----	538
Dynamena pumila                    TMRILEIKGSQV--LGKPQQEINFPGGK	545
Clytia hemisphaerica               TMRILSVEGDHH--IGTPESNIVFK---	539
Copula sivickisi                   TMRVLQVAHDGT-FIGKHDGVIRFA---	557
Chironex yamaguchii                TMRVLQVKSDGT-FIGKHDQEIRFG---	542
Haliclystus sanjuanensis           TVRILQVTEDAI-IGNENPADIKF----	541
Craterolophus convolvulus          TVRILQVTDDDV-IGRENKEDVRFSK--	543
Hydractinia symbiolongicarpus      TVRVLQVQEDK--IEGLGNPDITFPRV-	544
Atolla vanhoeffeni                 ----------------------------	271
Nanomia bijuga                     ----------------------------	107
Chrysaora quinquecirrha            TMRILQVSESGDVIGRRNEAEIFFT---	545
Cassiopea xamachana                TMRILQVSETGDVIGRRNEAEIYFS---	419
Rhopilema esculentum               TMRILQVSESGDVIGRRNEAEIFFS---	545
```

**SM4**. **Multiple alignment of Medusozoan pyruvate kinase (PK) protein sequences.**

The active-site signature is shaded in gray, and residues in black are also involved in the enzyme's active site (Enriqueta-Muñoz and Ponce, 2003; de Klerk et al., 2012). Accession numbers are not provided since sequences resulted from hits deposited as part of a Bioproject at NCBI.

```
cGAPDH      ------------------------------------------------------------	0
mGAPDH      MLRVVIGAGAFTLGAFGVVKTKELVDRTKSHDLKALDLVQCASAIGSTHLPLPSRHSLLN	60


cGAPDH      ----------MVIKVGINGFGRIG------------------------------------	14
mGAPDH      DLKANEEFDILVIGGGATGCGVALDAASRGLKTALVEKYDYACGTSSRSTKLIHGGVRYL	120
                      :**  * .* *

cGAPDH      ------------RLVCRAALEKSEIQIV--AINDPF--IDLEYMVYMFKYDS--------	50
mGAPDH      QKAIFNLDIEEYRLVKEALHERSNLLKVAPHLADPLPIMLPVYKLWQVPYFWAGIKMYDL	180
                        *** .*  *:*::  *   : **:  :   * :: . *

cGAPDH      -------------------------THKRFNGDIKTEDGK-------------------L	66
mGAPDH      VAGKRLVKNSYFVNKEKALDLFPMLKHENLRGAIIYYDGQHNDSRTNLALALTAIRKGAA	240
                                     .*:.:.* *   **:

cGAPDH      VI-----------------NGNKVFVYGKRNPE---EIPWSESGAEFVVESTGVFTTKEK	106
mGAPDH      CLNHVEVLHILKKTMKDENNENKEVVCGARVRDTATGDEWDVH-AKSVINATGPFTDNLR	299
             :                 * ** .* * *  :      *.   *: *:::** ** : :

cGAPDH      ASLHMKGGAKKVVISAPSADAPMFVMGVNEDKYDAANMHVV-------------------	147
mGAPDH      QMA--DEGTN--KICQPSAGVHIIL----PQYYSPKNMGLLDPATSDGRVIFFLPWEGAT	351
                 . *::   *. ***.. :::     : *.  ** ::

cGAPDH      ----SNASCTTNCLAPLAKIIHDNFGIEEA------------------------------	173
mGAPDH      IAGTTDSACDVT-FSPVPRESEVRFILDEIKHYLNNDVDVRRGDVLAAWSGIRPLVIDPS	410
                ::::* .. ::*: :  . .* ::*

cGAPDH      -----LMSTIHAVTATQKTVDGPSQKKWRDGRGAFQN-----------------------	205
mGAPDH      SKDTKSLSRNHVIEVSENKMITIAGGKWTTYRAMASDTVDTAIKTCNLSAGGSQTDGLML	470
                  :*  *.: .:::.:   :  **   *.  .:

cGAPDH      ----------------------------------IIPASTGAAKAVGKVIPDLNGKLT--	229
mGAPDH      EGGEGWTPTFFIRLVQDFGLEPEVAKHLAKSYGNKAPQVAKLAALTGKRWPVVGNRLVDD	530
                                                *  :  *  .**  * :..:*.

cGAPDH      ------GMAFRVPVPDVSVVDLTCRLKKP-----AKYDEIKAVVKKASESKELGKYMGYT	278
mGAPDH      FPYIEAEIRYGIREYALSAVDMIARRLRIAFLNVQAADEILP-----KVIKIMGEELGWD	585
                   : : :    :*.**: .*  :         ***       .  * :*: :*:

cGAPDH      DEEVVSSDFIGCTFSSIFDANAGIALSDNFVKLVAWYDNEYGYSHRVCDFIIH--MAK--	334
mGAPDH      EKKRSEELIMAR-----------------------KFLNTMGYRTRISAGDVPLDLSQND	622
            :::  .. ::.                         : *  **  *:.   :   :::

cGAPDH      ------------------------------------------------------------	334
mGAPDH      IDKYKRIFQNLDADNSGHSTIASLRLALEADRELNITNEQLDDLIRKFDLDASSPLDQEN	682


cGAPDH      ------------------------------------------	334
mGAPDH      FLRLVSALKAGPEANDSLGTLVQRQYDDVKLEDTSGIIFGGV	724
```

**SM5. The two GAPDH isoforms of the jellyfish *Stomolophus* sp.2.** cGAPDH indicates the cytosolic, and mGAPDH the mitochondrial enzyme. Both sequences share 24 % identity.

```
Chrysaora quinquecirrha   MAV------RKKLVDVLKNCQHSKCLLAPRPSSFVL--------KLSKYSS---------	37
Aurelia sp.1              MIFKHIGQASRMSVRQMPSWRPSCCNKLVSTQGLVSIQNEGLPQNGRRLFSK-LAKRSIT	59
Rhopilema esculentum      MEFRLAKKASPILS---QSWKPIYRRLSTTRSR----LCPLLQ--------KTKSRKSIP	45
Stomolophus sp.2          MALNNLKNTSRIIV---DVCKSYS----HRSVR----VTPGLSTNLRRFSGRRVMRRTRP	49
Acropora millepora        -------------------------------------MASFLRT-SIRPFRQ-VLDRRFL	21
Nematostella vectensis    -------------------------------------MRGLLAT-GQRLL------RSRD	16
Danio rerio               ----------------------------------MTLFRHLVRI-TS-------------	12
Bos taurus                -------------------------------------MAILLRP-AS-------------	9
Homo sapiens              -------------------------------------MARLLRS-AT-------------	9
Mus musculus              -------------------------------------MAMLLRV-AT-------------	9

Chrysaora quinquecirrha   AASSLGRVKLVSGNLKNAKNTRF----MSMSPAAVIEAIRPLKEICGEENVSTSLSVREH	93
Aurelia sp.1              KAYSMSAAMIQG-YEQGDFGRRY----KSMAPAAVVEAIQNLKSVCGEENVSTSMSVREH	114
Rhopilema esculentum      LLT---RPKFEQRQSGAILWKRN----NSMAPAAVIESLDSLRKICGAENVSTSMSVREH	98
Stomolophus sp.2          LIYNMTDTAQQYLTRAPVSGIRN----KSMAPAAIIEAINSLKEICGAENVSTSLSVREH	105
Acropora millepora        L------------PFCSVRWKSTVAQAKESYCEINDELKREFQEIVGEKNVSSALVVREQ	69
Nematostella vectensis    L------------CSRGRRWISTSHRKA---QTAVAALKPTMQTLLGEDNVSSAQAVRDQ	61
Danio rerio               --------------PRL-PFICGSSRRFSAKTAAVERVVSSFRSVTGDEGVSVGSAVREQ	57
Bos taurus                --------------WGLFSWRGYCS--RGTQGRLSAGFVEALKAVVGSPHVSTAAVVREQ	53
Homo sapiens              --------------WELFPWRGYCS--QKAKGELCRDFVEALKAVVGGSHVSTAAVVREQ	53
Mus musculus              --------------QRLSPWRSFCS--RGSQGGLSQDFVEALKAVVGSPHVSTASAVREQ	53
                                                                   :: : *   ** .  **::

Chrysaora quinquecirrha   HSHDESYHGGSSPAVVVFPTNVEQVSEIAKLCYKNNIHIIPFGTGTGLEGGVNATENGVC	153
Aurelia sp.1              HGHDESYHESMMPSAVLFPQNVEQVSEIAKLCYSKNIHMLPFGSGTGLEGGVSATEGGIS	174
Rhopilema esculentum      HSHDESYHESFMPNAVVFPQNVEQVSEVVKVCYSKNVHIIPFGTGTGLEGGVTATQGGIC	158
Stomolophus sp.2          HSHDESYHEPCMPSVVVFPQNVEQVSEIAKLCYKNAIHIVPFGTGTGLEGGVNATEDGVC	165
Acropora millepora        HGKDESHHTCQPADLVVWPENKEQVSQIAKLCNDQCIPLIPFGSGTGLEGGVVAKKGGVS	129
Nematostella vectensis    HGHDESYHGVMPPDLVVFPTSREQVSEIAKICYEHSVPMVPHGSGTGLEGGICAMKGGVC	121
Danio rerio               HGRDESVHRCRPPDVVVFPRSVEEVSALAKICHHYRLPIIPFGTGTGLEGGVGALQGGVC	117
Bos taurus                HGHDESMHRCQPPDAVVWPQNVEQVSRLAALCYGQGVPIIPFGTGTGLEGGVCAVQGGVC	113
Homo sapiens              HGRDESVHRCEPPDAVVWPQNVEQVSRLAALCYRQGVPIIPFGTGTGLEGGVCAVQGGVC	113
Mus musculus              HGHDESMHRCQPPDAVVWPQNVDQVSRVASLCYNQGVPIIPFGTGTGVEGGVCAVQGGVC	113
                          *.:*** *       *::* . ::** :. :*    : ::*.*:***:***: * :.*:.

Chrysaora quinquecirrha   IDLTKMDQIVDLHPEDFDVTVEPGVKRDALNHYIKDSGLWFPVDPGADCSLCGMAATSAS	213
Aurelia sp.1              IDLTKMDKITDLNPEDFDVTVEPGVKRSDLNHHVKDAGLWFPVDPGAECSLCGMVATSAS	234
Rhopilema esculentum      VDLTKMDKIIDLNPEDFDVTVEPGVKRDDLNHYVKDAGLWFPVDPGAECSLCGMVATSAS	218
Stomolophus sp.2          VDLTKMDKITDLNPDDFDVTVEPGVKREDLNHYVKDAGLWFPVDPGAECSLCGMVATSAS	225
Acropora millepora        VDVTKMNEILDFNPDDFDVTVQAGVSRLQLNNYVKDSGLWFPVDPGADASLGGMASTSAS	189
Nematostella vectensis    IDLSKMKTILEVNQEDFDTTVEAGVTREQLNAFIRDTGLWFPIDPGADASLCGMCATSAS	181
Danio rerio               FSLRKMEQVVDLHQEDFDVTVEPGVTRKSLNSYLRDTGLWFPVDPGADASLCGMAATSAS	177
Bos taurus                INLTRMDRILELNPEDFSVMVEPGVTRKALNTHLRDSGLWFPVDPGADASLCGMAATGAS	173
Homo sapiens              VNLTHMDRILELNQEDFSVVVEPGVTRKALNAHLRDSGLWFPVDPGADASLCGMAATGAS	173
Mus musculus              INLTHMDQITELNTEDFSVVVEPGVTRKALNTHLRDSGLWFPVDPGADASLCGMAATGAS	173
                          ..: :*. : :.: :**.. *: **.*  ** .::*:*****:****:.** ** :*.**

Chrysaora quinquecirrha   GTNAVRYGTMRENVINLEVVLADGTIINTAGKDRRTK-----------------------	250
Aurelia sp.1              GTNAVRYGTMRENVINLEVVLADGTIINTAGKDRRTK-----------------------	271
Rhopilema esculentum      GTNAVRYGTMRENVMNLEVVLADGKIIHTAGKDRRTK-----------------------	255
Stomolophus sp.2          GTNAVRYGTMRENVMNLEVVLADGTIIYTAGKGRRTK-----------------------	262
Acropora millepora        GTNAVRYGTMRENVMNLEVVLADGRIINTAGEGRRTR-----------------------	226
Nematostella vectensis    GTNAVRYGTMRENILNLEVVLSDGRVIDTAGKGRRTK-----------------------	218
Danio rerio               GTNAVRYGTMRENVLNLEVVLADGTILHTAGKGRRPR-----------------------	214
Bos taurus                GTNSVRYGTMRDNVLNLEVVLPSGQRLHTAGPGRRFR-----------------------	210
Homo sapiens              GTNAVRYGTMRDNVLNLEVVLPDGRLLHTAGRGRHFRFGFWPEIPHHTAWYSPCVSLGRR	233
Mus musculus              GTNAVRYGTMRDNVINLEVVLPDGRLLHTAGRGRHYR-----------------------	210
                          ***:*******:*::****** .*  : *** .*: :

Chrysaora quinquecirrha   KTAAGYNLTNLFVGSEGTLGIITKATIRLYPIPEMITAAVCNFPSIKDAVDCVVMILQCG	310
Aurelia sp.1              KTAAGYNLTNLFVGSEGTLGIVTKATLRLYPIPETIVAAVCSFPTIKDAVDCVVMILQCG	331
Rhopilema esculentum      KTAAGYNLTNLFVGSEGTLGLITKATLRLYPIPETIVAAVCSFPTIKDAVDSVVMILQCG	315
Stomolophus sp.2          KTAAGFNLTNLFVGSEGTLGLITKATLRLYPIPETIVAAVCSFPTIKDAVDCVVMILQCG	322
Acropora millepora        KTSAGYNLTNLFVGSEGTLGIITKVTLKLYGIPESTASGVSSFPDVKSAVDAVVEILQCG	286
Nematostella vectensis    KTSAGYNLTNLFAGSEGTLGIITKATLKLYGIPEATVSGVSSFPSVKSAVDATVEILQCG	278
Danio rerio               KTAAGYNLTNLFVGSEGTLGIITKATLRLYGVPESMVSAVCSFPSVQSAVDSTVQILQAG	274
Bos taurus                KSAAGYNLTGLFVGSEGTLGLITAATLRLHPVPEATVAATCAFPTVQAAVDSTVHILQAA	270
Homo sapiens              KSAAGYNLTGLFVGSEGTLGLITATTLRLHPAPEATVAATCAFPSVQAAVDSTVHILQAA	293
Mus musculus              KSAAGYNLTGLFVGSEGTLGIITSTTLRLHPAPEATVAATCAFPSVQAAVDSTVQILQAA	270
                          *::**:***.**.*******::* .*::*:  **  .:... ** :: ***..* ***..

Chrysaora quinquecirrha   IPIARIELMDDIAIDMCNKYSGTDYDVSPSLFLEFHGSDRSVEDQVEKTGEIVSMNEGSN	370
Aurelia sp.1              IPVARIELMDDVAMDMCNKYSSTDYHVAPSLFLEFHGSDRSVEDQVEKTNEIVTMNEGSN	391
Rhopilema esculentum      IPIARIELMDDLAMEMCNKYSGTDYTVAPSLFLEFHGSDKSVEDQLEKTNEIVNMNEGSN	375
Stomolophus sp.2          LPIARIELMDDVAMEMCNKYSGTDYAVTPSLFLEFHGSEKSVEDQLEKTNEIVNMNEGSN	382
Acropora millepora        LPMARIEFLDEVQIDACNKFSGLNYPVTPTLFLEFQGSENSVKEQAAVAADVIEGNGGSK	346
Nematostella vectensis    IPVARIEFLDDCMIDATNKYSGLDYPVQPTLFLEFTGSENSVEEQAITAGEVIAANEGSD	338
Danio rerio               VPIARIEFLDDVMINACNRFNNLSYAVTPTLFLEFHGSSKSMEEQVSVTEEITRDNGGSD	334
Bos taurus                VPVARIEFLDEVMMDACNRHSKLNCCVAPTLFLEFHGSEQALAEQVQRTEEIIRHNGGSH	330
Homo sapiens              VPVARIEFLDEVMMDACNRYSKLNCLVAPTLFLEFHGSQQALEEQLQRTEEIVQQNGASD	353
Mus musculus              VPVARIEFLDDVMMDACNRHSKLNCPVAPTLFLEFHGSQQTLAEQLQRTEAITQDNGGSH	330
                          :*:****::*:  ::  *:..  .  * *:***** **..:: :*   :  :   * .*.

Chrysaora quinquecirrha   FFWAKEMEERNRLWKARHSMYYAALAYRPGCKGVVTDVCVPISNLPELITCAKEDALASN	430
Aurelia sp.1              FFWAKEMEDRNRLWKARHSMYYAALAYKPGCKGIVTDVCVPVSHLPELVTAAKEDAISSN	451
Rhopilema esculentum      FFWAKEMEERNRLWKARHSMYYAALAFKPGCKGIVTDVCVPISNLPELVTAAKEDAVSSN	435
Stomolophus sp.2          FFWAKEMEERNRLWKARHSMYYAALAYKPGCKGVVTDVCVPISNLPDLVTAAKEDAVSSN	442
Acropora millepora        FQWATELEERNRLWKARHNAWYADMALRPGCKGVSTDVCVPISRLPEIIVETKADIAASN	406
Nematostella vectensis    FRWAKELEERNKLWKARHAAWYACLALRPGCKGVSTDVCVPISKLPEIIVETKEDIIASN	398
Danio rerio               FAWAEDEETRSRLWKARHDAWYAAMALRPGCKAYSTDVCVPISRLPQIIVETKADLISNN	394
Bos taurus                FSWAKEAEERSRLWAARHNAWYASLALRPGCKGYSTDVCVPISRLPEILVQTKEDLEAWR	390
Homo sapiens              FSWAKEAEERSRLWTARHNAWYAALATRPGCKGYSTDVCVPISRLPEIVVQTKEDLNASG	413
Mus musculus              FSWAKEAEKRNELWAARHNAWYAALALSPGSKAYSTDVCVPISRLPEILVETKEEIKASK	390
                          * ** : * *..** ***  :** :*  **.*.  ******:*.**:::. :* :  :

Chrysaora quinquecirrha   LLTPILGHVGDGNFHCFVTVDPNDPDEISEAKHFHERLARHALSLKGTCTGEHGIGLGKL	490
Aurelia sp.1              LITPILGHVGDGNFHCFVIMDPRDPDEVSAAKHFHERLARHALELNGTCTGEHGIGIGKM	511
Rhopilema esculentum      LVTPVLGHVGDGNFHCFVIVDPKDPDEISAAKHFHERLARHALSLNGTCTGEHGIGIGKM	495
Stomolophus sp.2          LITPFLGHVGDGNFHCFVIMDPKDHDEVSAAKHFHERLARHALSLNGTCTGEHGVGMGKM	502
Acropora millepora        ITAPIVGHVGDGNFHCFIPIKPGDEGELQTVKDFTMRLSRRALAMGGTCTGEHGIGNGKQ	466
Nematostella vectensis    LVAPIVGHVGDGNFHCFIVIDTSNEKEIQNAKDFTLRLGRRALAVGGTCTGEHGIGRGKL	458
Danio rerio               ITGPIAGHVGDGNFHCLIVLDPNDTDEVQRVHSFTERLARRALAMDGTCTGEHGIGLGKR	454
Bos taurus                LTGTIVGHVGDGNFHCILLVDPEDPEELLRVQAFAEQLGRRALALHGTCTGEHGIGLGKR	450
Homo sapiens              LTGSIVGHVGDGNFHCILLVNPDDAEELGRVKAFAEQLGRRALALHGTCTGEHGIGMGKR	473
Mus musculus              LTGAIVGHVGDGNFHCILLVDPDDAEEQRRVKAFAENLGRRALALGGTCTGEHGIGLGKR	450
                          :   . **********:: :.  :  *   .: *  .*.*:** : ********:* **

Chrysaora quinquecirrha    KLLKEEVGSETYNLMKTIKKSFDPKNILNPGKVLDFDL---------------------	528
Aurelia sp.1               KLLKEELGDNTINVMKSIKKALDPKNLLNPGKVLDYHY---------------------	549
Rhopilema esculentum       KLLQEELGHDTFNVMKQIKKALDPKNILNPGKVLDFYY---------------------	533
Stomolophus sp.2           KLLQEEIGDDTLNVMKLIKKSLDPKNLLNPGKVLNYHY---------------------	540
Acropora millepora         AMLPEEIGPTGLSVMKDIKSLLDPNWIMNPGKVFL------------------------	501
Nematostella vectensis     ALLEEEVGPSGIEVMKQIKQMLDPKNLMNPGKVLRM-----------------------	494
Danio rerio                ALLREEVGPLAIEVMKGLKASLDPRNLMNPGKVLELTQTNTEQ----------------	497
Bos taurus                 QLLPEEVGAVGMETMRQIKATLDPQGLMNPGKPEEGTVLRLQEEGHFRGPRSLSQLLCP	509
Homo sapiens               QLLQEEVGAVGVETMRQLKAVLDPQGLMNPGKVL-------------------------	507
Mus musculus               QLLQEEVGPVGVETMRQLKNTLDPRGLMNPGKVL-------------------------	484
                             :* **:*    . *: :*  :**. ::****
```

**SM6. Multiple alignment of animal mitD-iLDH protein sequences.** FAD-binding sites highlighted in grey (Jin et al., 2023). Residues marked with (*) identical residues; (:) conservative substitutions; and (.) semiconservative substitutions.

**SUPPLEMENTARY TABLES**

**ST1**. Reference protein sequences used for the phylogenetic analyses shown in Figures 6 and 7.

| **Species** | **Accession** | **Tree label** | **Database annotation** | **Database** |
| --- | --- | --- | --- | --- |
| *Bos taurus* | [Q148K4](https://www.uniprot.org/uniprotkb/Q148K4/entry) | *Bos taurus* | D-lactate dehydrogenase, mitochondrial | UniProt |
| *Canis lupus familiaris* | [XP852976.3](https://www.ncbi.nlm.nih.gov/protein/XP_852976.3?report=genpept) | *Canis lupus familiaris* | Probable D-lactate dehydrogenase, mitochondrial | GenBank |
| *Homo sapiens* | [Q86WU2](https://www.uniprot.org/uniprotkb/Q86WU2/entry) | *Homo sapiens* | D-lactate dehydrogenase, mitochondrial | UniProt |
| *Monodelphis domestica* | [XP001375142](https://www.ncbi.nlm.nih.gov/protein/XP_001375142) | *Monodelphis domestica* | probable D-lactate dehydrogenase, mitochondrial isoform X1 | GenBank |
| *Mus musculus* | [Q7TNG8](https://www.uniprot.org/uniprotkb/Q7TNG8/entry) | *Mus musculus* | D-lactate dehydrogenase, mitochondrial | UniProt |
| *Pan troglodytes* | [XP001139010](https://www.ncbi.nlm.nih.gov/protein/XP_001139010) | *Pan troglodytes* | D-lactate dehydrogenase, mitochondrial | GenBank |
| *Rattus norvegicus* | [Q7TPJ4](https://www.uniprot.org/uniprotkb/Q7TPJ4/entry) | *Rattus norvegicus* | Ac2-202 | UniProt |
| *Tetraodon nigroviridis* | [Q4T6R2](https://www.uniprot.org/uniparc/UPI00002F09A0/entry/Q4T6R2) | *Dichotomyctere nigroviridis* | D-lactate dehydrogenase (cytochrome) | UniProt |
| *Xenopus laevis* | [A1L2R0](https://www.uniprot.org/uniprotkb/A1L2R0/entry) | *Xenopus laevis* | D-lactate dehydrogenase, mitochondrial | UniProt |
| *Poeciliopsis prolifica* | [XP054884812](https://www.ncbi.nlm.nih.gov/protein/XP_054884812) | *Poeciliopsis prolifica* | Probable D-lactate dehydrogenase, mitochondrial isoform X2 | GenBank |
| *Aurelia* sp. 1 | [KX431209](https://www.ncbi.nlm.nih.gov/nuccore/KX431209) | *Aurelia* sp. 1 | Lactate dehydrogenase | GenBank |
| *Rhopilema esculentum* | [XP_065065346](https://www.ncbi.nlm.nih.gov/protein/XP_065065346) | *Rhopilema esculentum* | Probable D-lactate dehydrogenase, mitochondrial | GenBank |
| *Danio rerio* | [F1QXM5](https://www.uniprot.org/uniprotkb/F1QXM5/entry) | *Danio rerio* | D-lactate dehydrogenase, mitochondrial | UniProt |
| *Saccharomyces cerevisiae* | [P32891](https://www.uniprot.org/uniprotkb/P32891/entry) | *Saccharomyces cerevisiae* | D-lactate dehydrogenase [cytochrome] 1, mitochondrial | UniProt |
| *Kluyveromyces lactis* | [Q12627](https://www.uniprot.org/uniprotkb/Q12627/entry) | *Kluyveromyces lactis* | D-lactate dehydrogenase [cytochrome] 1, mitochondrial | UniProt |
| *Rhopilema esculentum* | [XP_065051904.1](https://www.ncbi.nlm.nih.gov/protein/XP_065051904.1) | *Rhopilema esculentum* | Creatine kinase, testis isozyme-like | GenBank |
| *Rhopilema esculentum* | [XP_065055456.1](https://www.ncbi.nlm.nih.gov/protein/XP_065055456.1) | *Rhopilema esculentum* | Creatine kinase B-type-like | GenBank |
| *Rhopilema esculentum* | [XP_065067122.1](https://www.ncbi.nlm.nih.gov/protein/XP_065067122.1) | *Rhopilema esculentum* | Creatine kinase, flagellar-like isoform X1 | GenBank |
| *Rhopilema esculentum* | [XP_065067123.1](https://www.ncbi.nlm.nih.gov/protein/XP_065067123.1) | *Rhopilema esculentum* | Creatine kinase, flagellar-like isoform X2 | GenBank |
| *Hediste diversicolor* | [P51546](https://www.uniprot.org/uniprotkb/P51546/entry) | *Hediste diversicolor* | Glycocyamine kinase | UniProt |
| *Alitta virens* | [Q8ISK0](https://www.uniprot.org/uniprotkb/Q8ISK0/entry) | *Alitta virens* | Glycocyamine kinase alpha subunit | UniProt |
| *Eisenia fetida* | [O15991](https://www.uniprot.org/uniprotkb/O15991/entry) | *Eisenia fetida* | Lombricine kinase | UniProt |
| *Thoracophelia sp. 1* | [A0A8D6MQ80](https://www.uniprot.org/uniprotkb/A0A8D6MQ80/entry) | *Thoracophelia* sp. | Opheline kinase | UniProt |
| *Actinia tenebrosa* | [XP_031557190.1](https://www.ncbi.nlm.nih.gov/protein/XP_031557190.1) | *Actinia tenebrosa* | Arginine kinase | GenBank |
| *Anthopleura japonica* | [O15992](https://www.uniprot.org/uniprotkb/O15992/entry) | *Anthopleura japonica* | Arginine kinase | UniProt |
| *Penaeus vannamei* | [Q004B5](https://www.uniprot.org/uniprotkb/Q004B5/entry) | *Penaeus vannamei* | Arginine kinase | UniProt |
| *Limulus polyphemus* | [P51541](https://www.uniprot.org/uniprotkb/P51541/entry) | *Limulus polyphemus* | Arginine kinase | UniProt |
| *Siphonosoma cumanense* | [Q4AED0](https://www.uniprot.org/uniprotkb/Q4AED0/entry) | *Siphonosoma cumanense* | Hypotaurocyamine kinase | UniProt |
| *Schistosoma mansoni* | [P16641](https://www.uniprot.org/uniprotkb/P16641/entry) | *Schistosoma mansoni* | Taurocyamine kinase | UniProt |
| *Hydra vulgaris* | [XP_065657321](https://www.ncbi.nlm.nih.gov/protein/XP_065657321) | *Hydra vulgaris* | Creatine kinase B-type | GenBank |
| *Hydra vulgaris* | [XP_065657547](https://www.ncbi.nlm.nih.gov/protein/XP_065657547) | *Hydra vulgaris* | Creatine kinase M-type-like isoform X1 | GenBank |
| *Hydra vulgaris* | [XP_065657548](https://www.ncbi.nlm.nih.gov/protein/XP_065657548) | *Hydra vulgaris* | Creatine kinase M-type-like isoform X2 | GenBank |
| *Hydra vulgaris* | [XP_065657549](https://www.ncbi.nlm.nih.gov/protein/XP_065657549) | *Hydra vulgaris* | Creatine kinase M-type-like isoform X2 | GenBank |
| *Homo sapiens* | [P32189](https://www.uniprot.org/uniprotkb/P32189/entry) | *Homo sapiens* | Glycerol kinase | UniProt |

**ST2.** The nuclear-encoded subunits from mitochondrial complexes not detected across the five representative Medusozoan datasets including the *C. sivickisi* transcriptome, *H. vulgaris* genome, *H. sanjuanensis* transcriptome, *R. esculentum* genome, and the *Stomolophus* sp.2 transcriptome.

| *Mitochondrial Complex* | *Nuclear encoded subunit* |
| --- | --- |
| *I* | NDUFS5 |
|  | NDUFV3 |
|  | NDUFA1 |
|  | NDUFA3 |
|  | NDUFAB1 |
|  | NDUFB2 |
|  | NDUFB3 |
|  | NDUFB4 |
|  | NDUFB5 |
|  | NDUFB6 |
|  | NDUFB11 |
|  | NDUFC1 |
|  | NDUFC2 |
| *III* | COR1  QCR8  QCR9  QCR10 |
| *IV* | COX17  COX8  COX7C  COX7B |
| *V* | k  j  f  e |

The protein components evaluated were defined based on the subunits present in the KEGG oxidative phosphorylation pathway (hsa00190 - *Homo sapiens*). No determined subunits indicate that no corresponding sequence was detected in the analyzed genomic or transcriptomic dataset and do not necessarily imply that the corresponding gene is absent from the organism.

**References**

Chen, Y., Zhang, Q., Hu, W. et al. (2017). Evolution and expression of the fructokinase gene family in Saccharum. BMC Genomics. 18:197. <https://doi.org/10.1186/s12864-017-3535-7>

de Klerk, N., de Vogel, C., Fahal, A., van Belkum, A., van de Sande, W.W.J. (2012). Fructose-bisphosphate aldolase and pyruvate kinase, two novel immunogens in Madurella mycetomatis. Med. Mycol. 50: 143–151. <https://doi.org/10.3109/13693786.2011.593005>

Enriqueta-Muñoz, M. and Ponce, E. (2003). Pyruvate kinase: current status of regulatory and functional properties. Comp. Biochem. Physiol. 135B: 197–218. <https://doi.org/10.1016/S1096-4959(03)00081-2>

Jin, S., Chen, X., Yang, J., Ding, J. (2023). Lactate dehydrogenase D is a general dehydrogenase for D-2-hydroxyacids and is associated with D-lactic acidosis. Nat. Commun. 14. 6638. <https://doi.org/10.1038/s41467-023-42456-3>

Kuser, P.R., Krauchenco, S., Antunes, O.A.C., Polikarpov, I. (2000). The high-resolution crystal structure of yeast hexokinase PII with the correct primary sequence provides new insights into its mechanism of action. J. Biol. Chem. 275: 20814–20821. <https://doi.org/10.1074/jbc.M910412199>

Poorman, R.A., Randolph, A., Kemp, R.G., Heinrikson, R.L. (1984). Evolution of phosphofructokinase- gene duplication and creation of new effector sites. Nature. 301(31): 467-469. <https://doi.org/10.1038/309467a0>

Shullia, N.I., Raffiudin, R., Juliandi, B. (2019). The phosphofructokinase and pyruvate kinase genes in Apis andreniformis and Apis cerana indica: exon-intron medusozoan and evolution. Trop. Life Sci. Res. 30: 89–107. <https://doi.org/10.21315/tlsr2019.30.1.6>
